# Integrated bioinformatic analysis of mitochondrial metabolism-related genes in acute myeloid leukemia

**DOI:** 10.3389/fimmu.2023.1120670

**Published:** 2023-04-17

**Authors:** Xiqin Tong, Fuling Zhou

**Affiliations:** Department of Hematology, Zhongnan Hospital of Wuhan University, Wuhan, China

**Keywords:** mitochondrial metabolism, tumor microenvironment, prognostic model, acute myeloid leukemia, drug sensitivity

## Abstract

**Background:**

Acute myeloid leukemia (AML) is a common hematologic malignancy characterized by poor prognoses and high recurrence rates. Mitochondrial metabolism has been increasingly recognized to be crucial in tumor progression and treatment resistance. The purpose of this study was to examined the role of mitochondrial metabolism in the immune regulation and prognosis of AML.

**Methods:**

In this study, mutation status of 31 mitochondrial metabolism-related genes (MMRGs) in AML were analyzed. Based on the expression of 31 MMRGs, mitochondrial metabolism scores (MMs) were calculated by single sample gene set enrichment analysis. Differential analysis and weighted co-expression network analysis were performed to identify module MMRGs. Next, univariate Cox regression and the least absolute and selection operator regression were used to select prognosis-associated MMRGs. A prognosis model was then constructed using multivariate Cox regression to calculate risk score. We validated the expression of key MMRGs in clinical specimens using immunohistochemistry (IHC). Then differential analysis was performed to identify differentially expressed genes (DEGs) between high- and low-risk groups. Functional enrichment, interaction networks, drug sensitivity, immune microenvironment, and immunotherapy analyses were also performed to explore the characteristic of DEGs.

**Results:**

Given the association of MMs with prognosis of AML patients, a prognosis model was constructed based on 5 MMRGs, which could accurately distinguish high-risk patients from low-risk patients in both training and validation datasets. IHC results showed that MMRGs were highly expressed in AML samples compared to normal samples. Additionally, the 38 DEGs were mainly related to mitochondrial metabolism, immune signaling, and multiple drug resistance pathways. In addition, high-risk patients with more immune-cell infiltration had higher Tumor Immune Dysfunction and Exclusion scores, indicating poor immunotherapy response. mRNA-drug interactions and drug sensitivity analyses were performed to explore potential druggable hub genes. Furthermore, we combined risk score with age and gender to construct a prognosis model, which could predict the prognosis of AML patients.

**Conclusion:**

Our study provided a prognostic predictor for AML patients and revealed that mitochondrial metabolism is associated with immune regulation and drug resistant in AML, providing vital clues for immunotherapies.

## Introduction

Acute myeloid leukemia (AML) is a common hematological malignancy characterized by poor prognosis and therapy resistance. The five-year survival rates of AML patients are less than 40% for patients aged 50 to 64 years and less than 10% for older patients (1). AML patients still receive the “7+3” chemotherapy protocol as their standard treatment for 50 years. The chemotherapy consists of a nucleoside analog and an anthracycline (2). Almost half of AML patients who have achieved complete remission still relapse (2). Although various promising novel therapies such as BCL-2 inhibitor venetoclax, IDH1 and IDH2 inhibitors (3), hypomethylating agents (4), antibody-drug conjugates (5), several FLT3 Tyrosine kinase inhibitors (TKIs) (6), and adoptive T cell therapies, have been approved or advancing through clinical trials in the past decade, patient responses to these novel therapies are not promising. For example, the leukemic cells with somatic mutations on the FLT3-TKD or BCR-ABL genes are resistant to TKIs treatment (7) or BCL2 inhibitor (8). Although mutation-targeted treatments have improved AML patient outcomes, many somatic mutations lack targeted treatment options, thus limiting their widespread clinical success (9). Therefore, it is necessary to study the unique traits of malignant leukemic cells independent of mutation to eradicate treatment-resistant leukemic cells.

Malignant cells maintain survival and rapid proliferation by altering their metabolic pathways to increase energy production (metabolic reprogramming) (10). Mitochondria are crucial bioenergetic hubs essential for tricarboxylic acid (TCA) cycle, fatty acid oxidation, the electron transport chain (ETC), and oxidative phosphorylation (OXPHOS) processes. These processes may promote malignant phenotype development and maintenance. Mitochondria also produce reactive oxygen species, which could be pro-tumorigenic (11). Although Warburg described that malignant cells produce energy mainly *via* aerobic glycolysis, many studies have demonstrated that many tumor cells depend on mitochondrial oxidative metabolism to obtain energy (12–14), indicating that inhibiting glycolysis does not prevent tumor proliferation. OXPHOS maintains leukemia stem cells (LSCs) and influences treatment resistance in AML (15). Additionally, Wu et al. reported that the mitochondria related genes were upregulated in AML and related to poor prognosis (16). Moreover, mitochondrial metabolism is crucial for tumor microenvironment (TME), and the differentiation and activity of immune cells, such as macrophages (17) and T cells (18). Changes in mitochondrial function can affect immunotherapy effectiveness (19–21). These findings indicate that mitochondrial metabolism is crucial for tumor biological activity. Therefore, it is necessary to identify specific energy metabolism dependencies and how they relate to the cellular microenvironment to provide novel treatment options for AML patients.

A recent study constructed a metabolism-related prognostic model in AML, consisting of FADS1, NEU1, SLC2A5, TBXAS1 and PDE4B (22). However, this study didn’t explore the relationship of TME and metabolism. In addition, another study found that four mitochondria-related genes (HPDL, CPT1A, IDH3A, and ETFB) influence the prognosis and BM microenvironment of AML patients (23). However, the bioinformatics study was validated based on a children cohort and relatively simple: no omics integration analysis, no somatic mutation assessment, or without experimental validation. Our study aimed to identify molecular targets and therapeutic mechanisms related to AML.

## Materials and methods

### Data acquisition

Public transcriptome and clinical data were collected from the Cancer Genome Atlas (TCGA) and the Gene Expression Omnibus (GEO) database (24) up to May 28, 2022. Three eligible AML cohorts (TCGA-LAML, GSE12714 (25), and GSE37642 (26)) were selected for further investigation. Patients without survival data were excluded. For the TCGA dataset, the RNA transcriptome data were acquired from the Genomic Data Commons (GDC) database *via* the TCGAbiolinks package (27) and normalized to fragments per kilobase of exon model per million mapped fragments (FPKM) values, while the clinical data were acquired *via* the UCSC Xena database (28). For GEO datasets, GEOquery package was used to obtain the normalized matrix files and clinical data (29). The baseline information of the included patients is summarized in **Table S1**.

MMRGs and immune-related genes were obtained from GeneCards database (30). A total of 50 MMRGs were obtained with a relevance score >5 (**Table S2**), and 27 immune-related genes were obtained with a relevance score >20. Twenty DNA repair genes were also obtained from published literature (31) (**Table S3**).

### Genomic analysis

Somatic mutation profile of AML patients was obtained from the TCGA database. Mutation signatures of MMRGs were summarized and presented using the R package Maftools (32). The chromosomal locations of MMRGs were presented using the R package RCircos (33).

### Generation of mitochondrial metabolism scores (MMs)

The relative abundance of a gene set enriched in a sample is widely detected using the single sample gene set enrichment analysis (ssGSEA) algorithm. In this study, MMs of patients in the three AML datasets were calculated using GSVA package (34) through the ssGSEA algorithm based on the expression data of MMRGs. The patients in the three datasets were separated into high- and low-MMs groups based on the cut-off values. Cut-off values with the lowest significant (P-value< 0.05) log-rank P-value were chosen as the optimal cut-off values (35). Two-group comparisons were analyzed with Wilcoxon rank sum test. One-way ANOVA and Tukey’s Honestly Significance Difference test were used to compare MMs among different French–American–British (FAB) subgroups.

### Weighted co-expression network analysis (WGCNA)

First, the limma package was used to determine the differentially expressed genes (DEGs) between high- and low-MMs groups in the TCGA-LAML dataset (36). DEGs with the adjusted P-value< 0.05 and |logFC|> 0 were included for analysis. The DEGs were used for WGCNA *via* the WGCNA package (37) to determine the gene modules related to MMs with highly correlated expression profiles across samples. WGCNA was run with networkType = “signed”, minModuleSize = 30, and softpower = 21.

### Unsupervised clustering for 17 MMRGs

The patients were classified in TCGA-LAML datasets using the R package ConsensusClusterPlus (38) based on the expression of MMRGs *via* the consensus clustering algorithm (39). The cluster size was set between 2 and 8. About 80% of the samples were randomly selected 50 times to ensure classification stability. Wilcoxon rank-sum test was used to determine the expression differences of MMRGs between different subtypes of AML patients. P-value< 0.05 was considered statistically significant.

### Construction of the prognosis model with MMRGs

The MMRGs with prognostic value (P-value< 0.1) were selected among the 17 MMRGs *via* univariate Cox analysis for the least absolute and selection operator (LASSO) regression. LASSO regression was performed *via* the glmnet package (40) based on prognostic MMRGs with parameter family = “cox” and ten-fold cross-validation, and repeated 1,000 times to prevent overfitting. A prognosis model was built *via* the multivariate Cox regression based on the MMRGs selected through LASSO regression. Per-patient risk score was calculated based on the prognostic model formula.


$$\text{RiskScore }=\sum_{i}\text{Coefficient }\left(\text{key gene}i\right)*\text{mRNA expression }\left(\text{key gene}i\right)$$

Patients were categorized into high- and low-risk groups based on the optimal cut-off value. An internal verification nomogram for overall survival (OS) prediction was developed using the rms package to verify the prognostic accuracy of the model. Additionally, calibration curves and decision curve analysis (DCA) were used to verify the model’s performance (41). Further external validation was performed using the GSE12417 cohort.

### Immunohistochemistry (IHC) and hematoxylin-eosin staining (H&E)

Bone marrow specimens were fixed in paraformaldehyde, decalcified, and embedded in paraffin. Tissue sections were deparaffinized and rehydrated, and then stained with H&E, or incubated with antibodies, specifically ECHS1 (Cat No. 11305-1-AP; proteintech, China), and NUDFS2 (Cat No. R27071; Zenbioscience, China). Antigens were detected using diaminobenzidine. Images were captured using a microscope equipped with a SPOT camera under high-power fields (400x). The mean intensity optical density (IOD) was used to quantify the immunohistochemical expression measured by ImageJ. This study was performed according to the Declaration of Helsinki, and received approval from the Ethics Committee of Zhongnan Hospital of Wuhan University.

### Identification and enrichment analysis of differentially expressed MMRGs

The limma package was used to determine the DEGs between high- and low-risk groups of the TCGA-LAML cohort (36) for further analysis. Adjusted P-value<0.05 and |logFC| >1 were set as the inclusion criteria.

Gene Ontology (GO) analysis (42), Kyoto Encyclopedia of Genes and Genomes (KEGG) enrichment analysis (43), and Disease Ontology (DO) analysis (44) were performed using the clusterProfiler package to reveal the characteristics of the DEGs (45). Besides, Gene Set Enrichment Analysis (GSEA) (46) was performed using the clusterProfiler package (45). The c2.all.v7.5.1.entrez.gmt gene set was acquired *via* the Molecular signatures database 3.0 (MSigDB) (47) as the reference gene set. Significantly enriched pathways were determined at P-value< 0.05 and FDR< 0.25.

### Construction of Protein-protein interaction (PPI) network and hub gene identification

PPI networks were constructed *via* the STRING database based on the DEGs (48) (confidence level 0.400) and visualized using Cytoscape software. MCC (Matthews Correlation Coefficient metric) (49), MNC (the maximal neighbourhood coefficient), and Degree algorithms were used to identify the hub genes.

### Immune infiltration estimation and correlation analysis

Tumor immune activity in AML patients was assessed using the ESTIMATE package based on TCGA-LAML expression profiles (50). The Tumor Immune Dysfunction and Exclusion (TIDE) immune scores were estimated through the TIDE database (51) to predict patients’ immunotherapy responses. Also, the infiltration abundance of 22 immune cells was determined using the CIBERSORTx database (52) based on patients’ transcriptome data. Besides, enrichment scores of 28 tumor-infiltration-associated immune cells were calculated using the ssGSEA algorithm in the GSVA package to represent the immune infiltration levels (53) in each sample. The correlation of different immune cells within two groups was calculated using the spearman algorithm. The expression matrix of the TCGA-LAML dataset was combined to calculate correlations between immune cells and hub genes in different groups. The correlation heat map was developed through the pheatmap package.

### Construction of mRNA-RNA binding proteins (RBPs) and mRNA-drugs interaction networks

RBPs interacting with hub genes were predicted using ENCORI database (54). mRNA-RBPs interaction pairs were then screened with clipExpNum > 2 and clipIDnum > 2 as screening criteria and plotted mRNA-RBPs interaction network. In addition, potential drugs or small molecule compounds interacting with hub genes were predicted using the drug-gene interaction database (DGIdb) (55). Cytoscape software was used to visualize mRNA-RBPs and mRNA-drugs interaction networks.

### Drug sensitivity analysis of hub genes

The relationships between hub genes and drug sensitivity were explored based on their transcriptome profiles and drug-sensitive profiles downloaded from the Genomics of Drug Sensitivity in Cancer (GDSC) database (56), the Cancer Cell Line Encyclopedia (CCLE) database (57), and the CellMiner database (58).

### Statistical analysis

R software (Version 4.1.2) was used for data processing and statistical analyses. Students t-test and Wilcoxon rank sum test were used for two-group comparisons, while Kruskal-Wallis tests were used for three or more group comparisons. Chi-square or Fisher’s exact tests were used to compare categorical variables. The survival package was used to perform survival analysis. Kaplan-Meier (KM) curves were used to express the survival difference between the two groups, and its significance was measured using a log-rank test. Spearman correlation analysis was used to calculate the correlation of distinct variables if not specified. The P-value was adjusted by the Benjamini and Hochberg method.

## Results

### Mutation profile of MMRGs and construction of MMs

**Figure 1:**
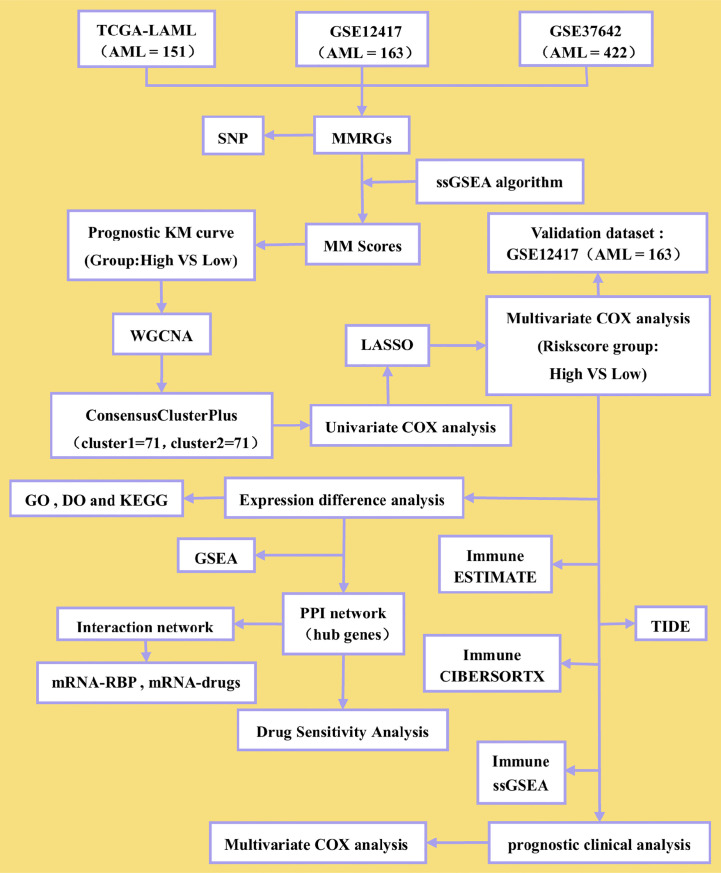
Flow diagram.

**Figure 2:**
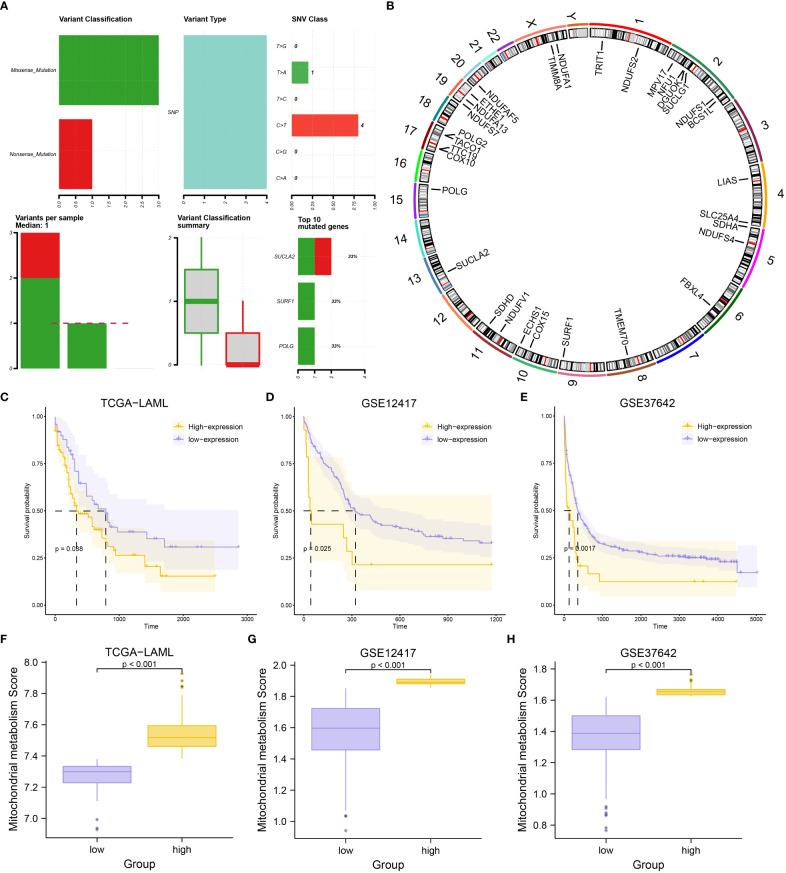
Mutational analysis of MMRGs in AML patients and construction of MMs. **(A)** The mutational landscape of MMRGs in AML. **(B)** Localization of MMRGs on chromosomes. **(C-E)** KM curves for the TCGA-LAML dataset **(C)**, GSE12417 dataset **(D)**, and GSE37642 dataset **(E)** grouped by high and low MMs. **(F-H)** Comparison of MMs between high- and low-MMs groups in the TCGA-LAML dataset **(F)**, GSE12417 dataset **(G)**, and GSE37642 dataset **(H)**.

A general overview of the study is presented in **Figure 1**. The three datasets (TCGA-LAML, GSE12417, and GSE37642) were first normalized using the limma package, with the TCGA-LAML dataset containing 151 samples (**Figures S1A, B**), the GSE12417 dataset containing 163 samples (**Figures S1C, D**), and the GSE37642 dataset containing 422 samples (**Figures S1E, F**). The expression profiles of the three datasets were consistent among samples after normalization (**Figures S1A, F**). The mutation profile of the 31 MMRGs in AML samples was analyzed to evaluate the mutation characteristic of the 31 MMRGs (BCS1L, COX10, COX15, DGUOK, ECHS1, ETHE1, FBXL4, LIAS, MPV17, NDUFA1, NDUFA13, NDUFAF5, NDUFS1, NDUFS2, NDUFS4, NDUFS7, NDUFV1, NFU1, POLG, POLG2, SDHA, SDHD, SLC25A4, SUCLA2, SUCLG1, SURF1, TACO1, TIMM8A, TMEM70, TRIT1, TTC19). The MMRGs had few single nucleotide polymorphisms (SNPs) in patients in the TCGA-LAML cohorts, only three genes (SUCLA2, SURF1, and POLG) with somatic mutations among 31 MMRGs in three AML patiens (**Figure 2A**, **Figure S2A**), mainly missense mutation. Single nucleotide variant (SNV) with C>T was the most prevalent, followed by T>A (**Figure 2A**).

RCircos package was used to annotate the chromosome location of 31 MMRGs to analyze the location of the 31 MMRGs on human chromosomes (**Figure 2B**). The MMRGs were mainly distributed in the 2, 5, 10, 11, and 17 chromosomes, of which most were distributed on the second chromosome (6 MMRGs) (**Figure 2B**). MMRGs located on the same chromosome were closely related at the genome level.

To investigate the overall expression of MMRGs in AML, MMs of each patient were calculated to represent the mitochondrial metabolism levels of AML patients. The patients were divided into two groups after combining the prognostic information and MMs of AML patients in the three datasets (excluding the AML patients lacking OS and OS. time) based on the MMs levels. Patient survival outcomes were significantly different per grouping (P-value< 0.05) depending on the best cut-off values for three datasets; TCGA-LAML (P-value = 0.038, **Figure 2C**), GSE12417(P-value = 0.025, **Figure 2D**), GSE37642 (P-value = 0.0017, **Figure 2E**). Moreover, the levels of MMs were significantly different between the two groups in the three datasets (P-value< 0.001) (**Figures 2F–H**). What’s more, we found that MMs constructed based on the expression of 31 MMRGs in the TCGA-LAML dataset could accurately diagnose the M0-M5 stages of AML patients (P-value< 0.05, **Figure S2B**, **Table S4**).

### Identification of co-expression modules through WGCNA

**Figure 3:**
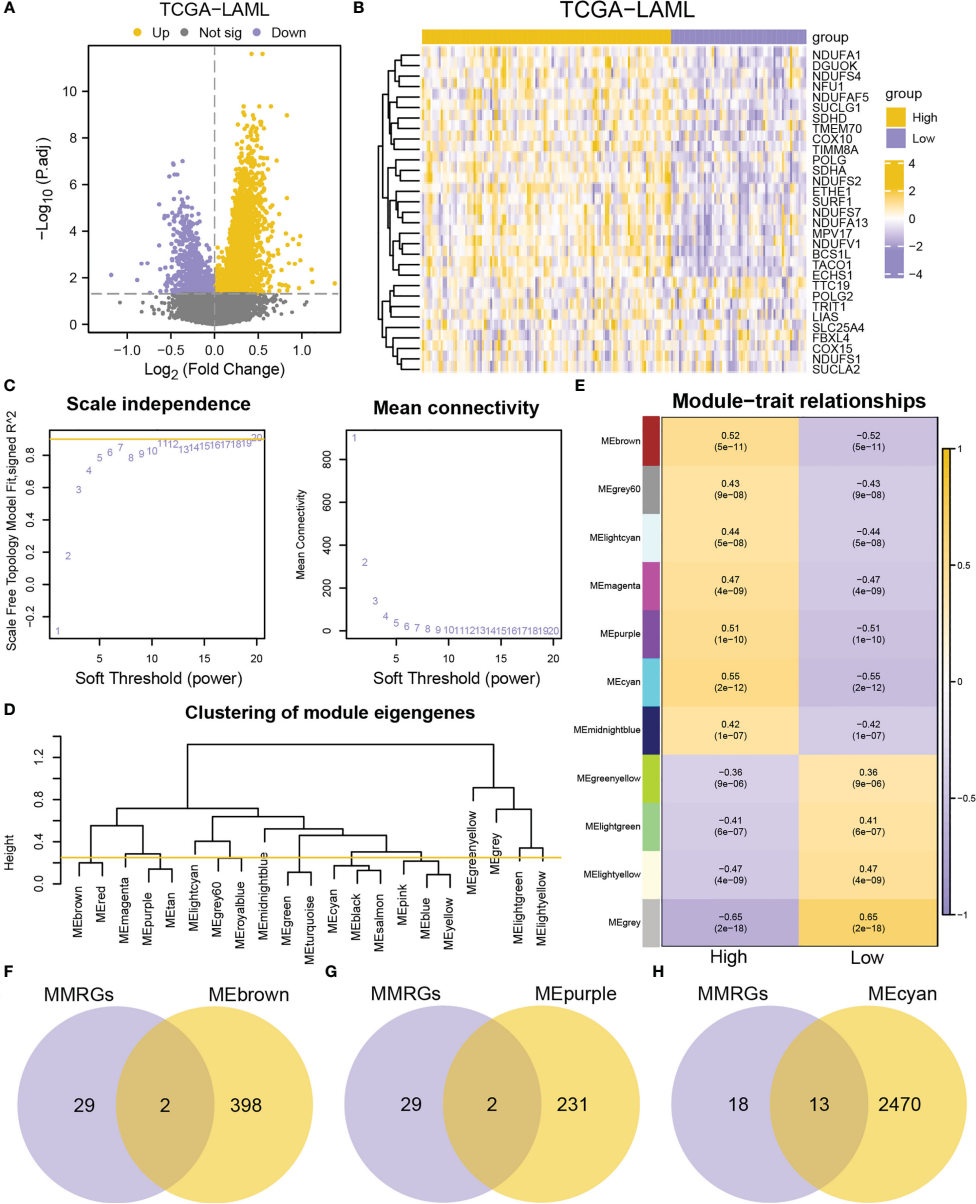
Identification of co-expression modules by WGCNA. **(A)** Volcano plot of DEGs between high- and low-MMs groups in the TCGA-LAML dataset. **(B)** Heatmap of MMRGs in the TCGA-LAML dataset. **(C)** The mean connectivity and scale free topology module fit for different soft-threshold powers. **(D)** A cluster dendrogram of module eigengenes. **(E)** Relationships between the module eigengenes and MMs. **(F-H)** Venn diagrams of MMRGs with MEbrown **(F)**, MEpurple **(G)**, and MEcyan **(H)**.

A total of 3,798 DEGs were identified (|logFC| > 0, adjusted P-value< 0.05) (3,036 upregulated and 762 downregulated genes) in the high-MMs group (**Figure 3A**). Furthermore, the expressions of the MMRGs were significantly different in the TCGA-LAML dataset (**Figure 3B**). To obtain genes closely associated with mitochondrial metabolism, WGCNA were conducted to identified modules in which genes were highly correlated. The modules with similarity lower than 0.25 (**Figure 3D**) were merged to obtain 11 modules at the scale independence of 0.9 (**Figure 3C**) (MEbrown, MEgrey60, MElightcyan, MEmagenta, MEpurple, MEcyan, MEmidnightblue, MEgreenyellow, MElightgreen, MElightyellow, and MEgrey). The correlations between the 11 module eigengenes and different MMs groups were then investigated (**Figure 3E**). The genes of the MEbrown (|r|=0.52, P-value =5e^-11^), MEpurple (|r|=0.51, P-value =1e^-10^), and MEcyan (|r|=0.55, P-value =2e^-12^) modules with correlation coefficients greater than 0.5 were selected for the analysis. The 31 MMRGs were intersected with genes in MEbrown (**Figure 3F**), MEpurple (**Figure 3G**) and MEcyan (**Figure 3H**) modules to obtain the 17 MMRGs modules (BCS1L, COX10, DGUOK, ECHS1, ETHE1, MPV17, NDUFA1, NDUFA13, NDUFS2, NDUFS7, NDUFV1, POLG, SDHA, SUCLG1, SURF1, TACO1, and TIMM8A).

Additionally, the correlations between the 17 MMRGs modules in the TCGA-LAML cohorts were explored. The 17 MMRGs modules were significantly correlated according to the TCGA-LAML dataset (**Figure S3A**). BCS1L and NDUFV1 (r = 0.701, P-value< 0.001, **Figure S3B**), MPV17 and NDUFV1 (r = 0.645, P-value< 0.001, **Figure S3C**), DGUOK and NDUFA1 (r = 0.653, P-value< 0.001, **Figure S3D**), NDUFA13 and NDUFS7 (r = 0.681, P-value< 0.001, **Figure S3E**), NDUFS7 and NDUFV1 (r = 0.643, P-value< 0.001, **Figure S3F**) were the top five pairs of genes with the highest correlations (**Figures S3B–F**).

### Unsupervised clustering for MMRGs

**Figure 4:**
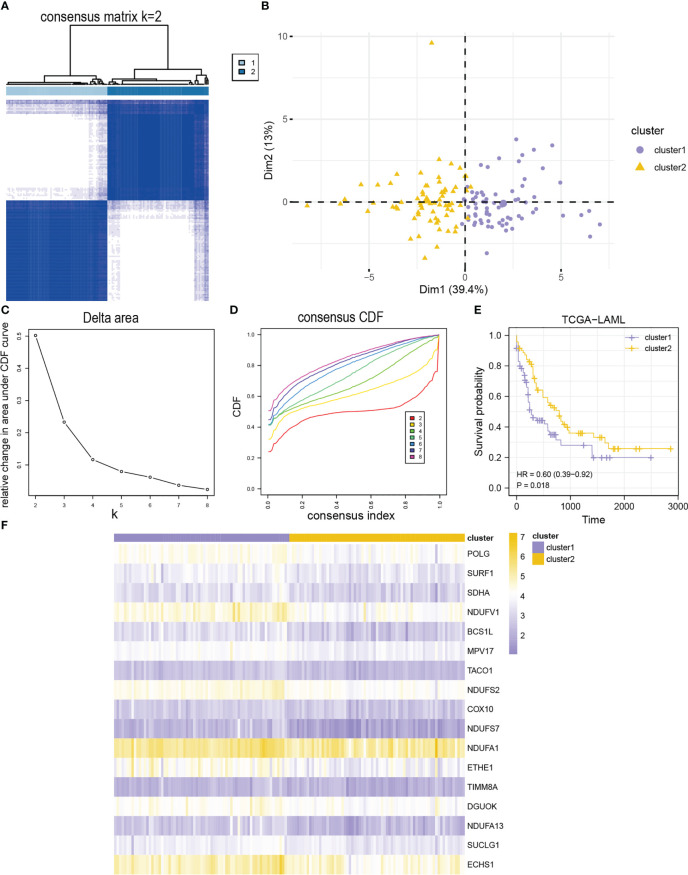
Identification of AML subtypes based on 17 module MMRGs. **(A)** The consensus clustering matrix of the TCGA-LAML cohort for K = 2. **(B)** Principal component analysis for the expression profiles of two AML clusters in the TCGA-LAML dataset. **(C, D)**. Relative changes in the area under cumulative distribution function (CDF) curve **(C)**, and consensus clustering CDF for k = 2-8 **(D)**. **(E)** KM curves of two AML clusters in the TCGA-LAML cohort. **(F)** Heatmap of 17 module MMRGs in the two AML clusters.

Two AML subtypes (cluster1 and cluster2) were identified based on the expression of the MMRGs modules *via* an unsupervised clustering method in the TCGA-LAML to investigate differential expression of the MMRGs modules in AML patients (**Figure 4A**). Cluster1 and cluster2 contained 71 samples and 71 samples, respectively. Principal component analysis (PCA) showed that the two AML subtypes had significant differences (**Figure 4B**). The consensus clustering had the best stability when k = 2 (**Figures 4C, D**). Subsequent survival analysis for the two AML clusters indicated that the clinical outcomes of two AML subtypes were significantly different (P-value = 0.018, **Figure 4E**). Furthermore, the expression levels of 17 MMRGs modules were significantly different between the two AML subtypes (**Figure 4F**).

Furthermore, the MMRGs modules were significantly upregulated in cluster1 in the TCGA-LAML dataset (**Figure S4A**). The expression of COX10 and TIMM8A was significantly different between the two AML subtypes (P-value< 0.01). However, the expression of the other 15 MMRGs was significantly different between the two AML subtypes (P-value< 0.001). The ROC curves showed that expression levels of COX10 (AUC = 0.650, **Figure S4C**), NDUFA1 (AUC = 0.673, **Figure S4H**), and TIMM8A (AUC = 0.630, **Figure S4R**) had lower accuracy in distinguishing two AML subtypes, while the expression levels of BCS1L (**Figure S4B**), DGUOK (**Figure S4D**), ECHS1 (**Figure S4E**), ETHE1 (**Figure S4F**), MPV17 (**Figure S4G**), NDUFA13 (**Figure S4I**), NDUFS2 (**Figure S4J**), NDUFS7 (**Figure S4K**), NDUFV1 (**Figure S4**L), POLG (**Figure S4M**), SDHA (**Figure S4N**), SUCLG1 (**Figure S4O**), SURF1 (**Figure S4P**), and TACO1 (**Figure S4Q**) could accurately distinguish the two AML subtypes (**Figures S4B–R**).

### Establishment and validation of MMRGs prognosis model

**Figure 5:**
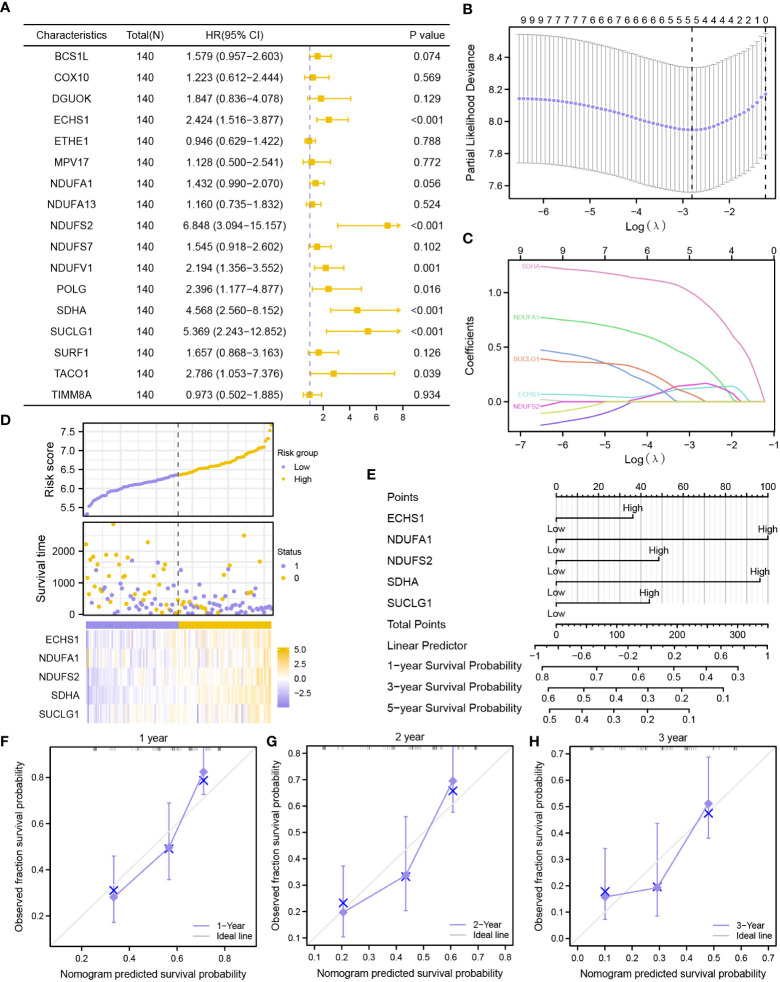
Construction of a prognostic risk signature based on MMRGs. **(A)** The forest plot showing the univariate Cox regression results of 17 module MMRGs in the TCGA-LAML dataset. **(B, C)**. Partial likelihood deviance of different numbers of variables **(B)**, and coefficient profiles **(C)** for the LASSO regression model. **(D)** Distribution of risk scores and survival status, and expression of five MMRGs. **(E)** A nomogram for multivariate Cox regression analysis of prognostic MMRGs. **(F-H)**. Calibration curves of the MMRGs prognostic model for 1-, 2-, and 3-year outcomes.

First, nine MMRGs modules correlated with the survival outcomes with P-value< 0.1 were selected *via* univariate Cox regression analysis (**Figure 5A**, **Table S5**) for further analysis. Finally, only five MMRGs modules (ECHS1, NDUFA1, NDUFS2, SDHA, SUCLG1) were selected through the LASSO regression analysis with minimized lambda (**Figures 5B, C**) for the construction of the prognosis model (**Figure 5D**).

Further validation of the prognostic value of the five MMRGs was conducted by analyzing the survival information of AML patients in TCGA-LAML dataset (**Table 1**). A prognostic model was then constructed based on the expression of five MMRGs using multivariate Cox regression (**Table 2**).

**Table 1:** Patient Characteristics of LAML patients in the TCGA datasets.

Characteristic	levels	Overall
n		151
Gender, n (%)	Female	68 (45%)
	Male	83 (55%)
Race, n (%)	Asian	1 (0.7%)
	Black or African American	13 (8.7%)
	White	135 (90.6%)
Age, n (%)	<=60	88 (58.3%)
	>60	63 (41.7%)
BM blasts (%), n (%)	<=20	60 (39.7%)
	>20	91 (60.3%)
OS event, n (%)	Alive	54 (35.8%)
	Dead	97 (64.2%)
Age, median (IQR)		56 (42, 66.5)

**Table 2:** Cox regression to prognosis MMRGs associated with OS in TCGA-LAML.

Characteristics	Total(N)	Univariate analysis		Multivariate analysis	
		Hazard ratio (95% CI)	P value	Hazard ratio (95% CI)	P value
ECHS1	140	2.424 (1.516-3.877)	<0.001	0.960 (0.513-1.797)	0.900
NDUFA1	140	1.432 (0.990-2.070)	0.056	1.777 (0.950-3.324)	0.072
NDUFS2	140	6.848 (3.094-15.157)	<0.001	1.274 (0.376-4.314)	0.697
SDHA	140	4.568 (2.560-8.152)	<0.001	3.767 (1.753-8.095)	<0.001
SUCLG1	140	5.369 (2.243-12.852)	<0.001	1.520 (0.514-4.501)	0.449

**Figure 6:**
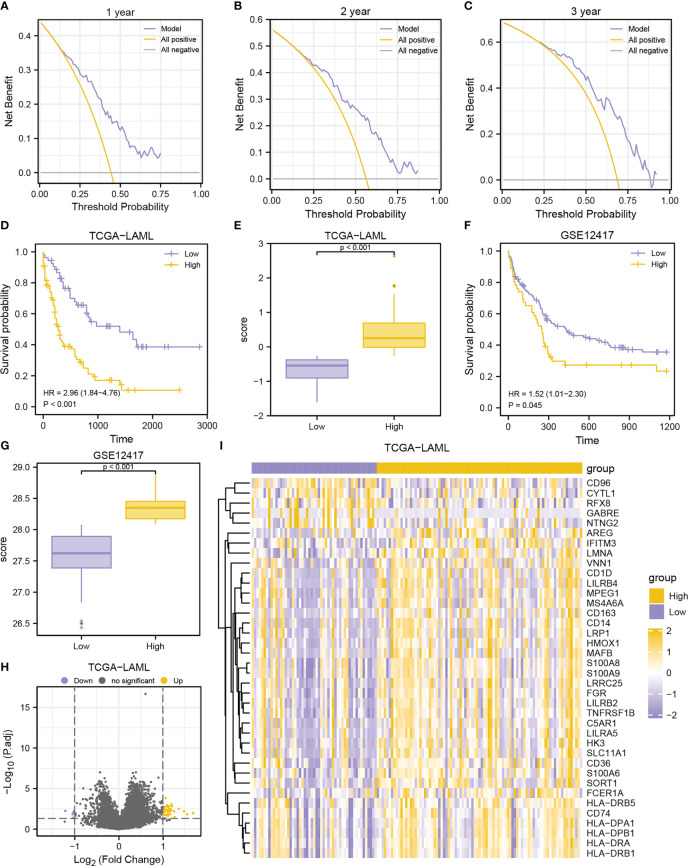
Prognostic performance of the MMRGs prognosis model. A-C. DCA curves of MMRGs prognosis model for the 1-year **(A)**, 2-year **(B)**, and 3-year **(C)**. D-E. Prognostic KM curves **(D)** and group comparison **(E)** in TCGA-LAML dataset. **(F, G)**. Prognostic KM curves **(F)** and group comparison **(G)** in GSE12417 cohort. **(H, I)**. Volcano plot **(H)** and heatmap **(I)** of DEGs between high- and low-risk groups in TCGA-LAML cohort.

The prognosis model of the MMRGs was evaluated using nomogram analysis (**Figure 5E**). The expression of NDUFA1 and SDHA performed better significantly in the Cox regression model than other variables. In addition, the nomogram performed best at predicting 1-year OS based on calibration plots (**Figures 5F–H**). DCA at 1-year (**Figure 6A**), 2-year (**Figure 6B**), and 3-year (**Figure 6C**) were performed to assess the clinical applicability of the nomogram. The results indicated that the nomogram had a higher net clinical benefit at 1-year. The KM curve showed that low-risk patients had markedly longer survival times than other patients (P-value< 0.001, **Figure 6D**). In addition, risk scores of two groups were significantly different (P-value< 0.001, **Figure 6E**).

Further assessment was conducted to assess the predictive capability of prognosis model in the GSE12417 dataset (**Figures 6F, G**). Similarly, high-risk patients had worse outcomes (P-value = 0.045, **Figure 6F**) and higher risk scores (P-value< 0.001, **Figure 6G**) in the GSE12417 dataset, indicating that the prognosis model developed by MMRGs could accurately predict the survival outcomes of AML patients.

DEGs were obtained *via* differential analyses on the TCGA-LAML dataset. A total of 38 DEGs were identified (|logFC| > 1, adjusted P-value< 0.05) (33 upregulated and 5 downregulated genes) in the high-risk group displayed in the volcano plot and heatmap (**Figures 6H–I**).

### Validation of ECHS1 and NDUFS2 by IHC

**Figure 7:**
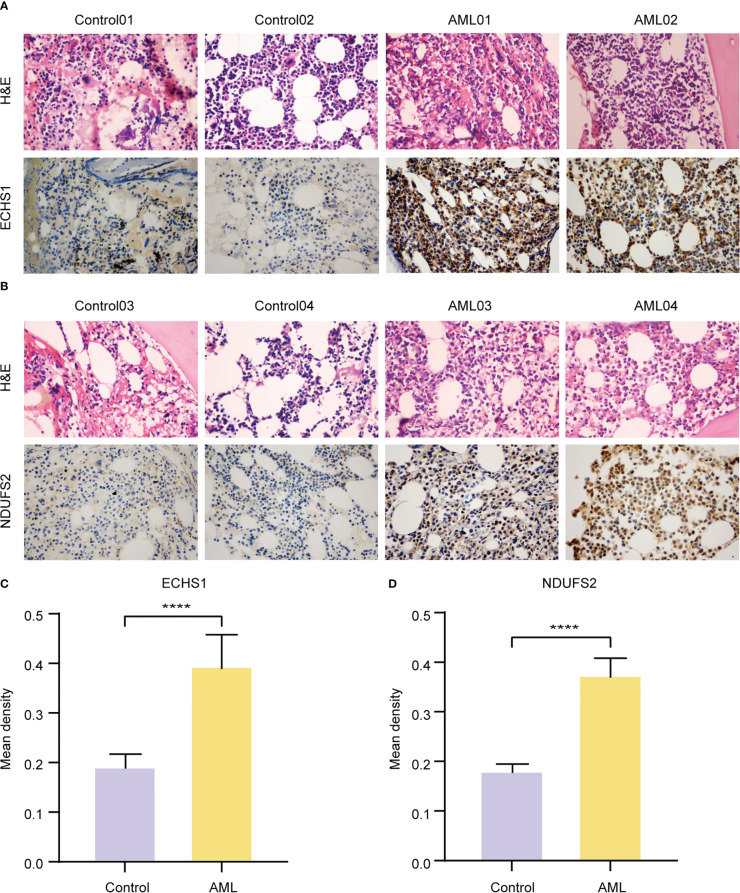
Validation of the expression levels of key MMRGs by IHC. A-B. H&E and ECHS1 **(A)** and NDUFS2 **(B)** IHC staining of BM samples from nonneoplastic patients and AML patients. Original magnification, 400X. **(C-D)**. Statistics of ECHS1 **(C)** and NDUFS2 **(D)** mean IOD from IHC images of nonneoplastic patients (n = 10) and AML patients (n = 10). ****P-value< 0.0001.

Expressions of ECHS1 and NDUFS2 were analyzed by IHC staining in bone marrow (BM) biopsies of 10 AML patients, and 10 non-neoplastic patients. Control BM samples showed weak ECHS1 (**Figure 7A**) and NDUFS2 expression (**Figure 7B**), and most cells were negative. However, most cells showed strongly positive signals of ECHS1 and NDUFS2 in AML samples (**Figures 7A, B**). According to the statistics of mean IOD from IHC images, AML samples had significantly increased expression levels of ECHS1 (**Figure 7C**) and NDUFS2 (**Figure 7D**) when compared to normal BM samples.

### Functional annotation of MMRGs phenotypes

**Figure 8:**
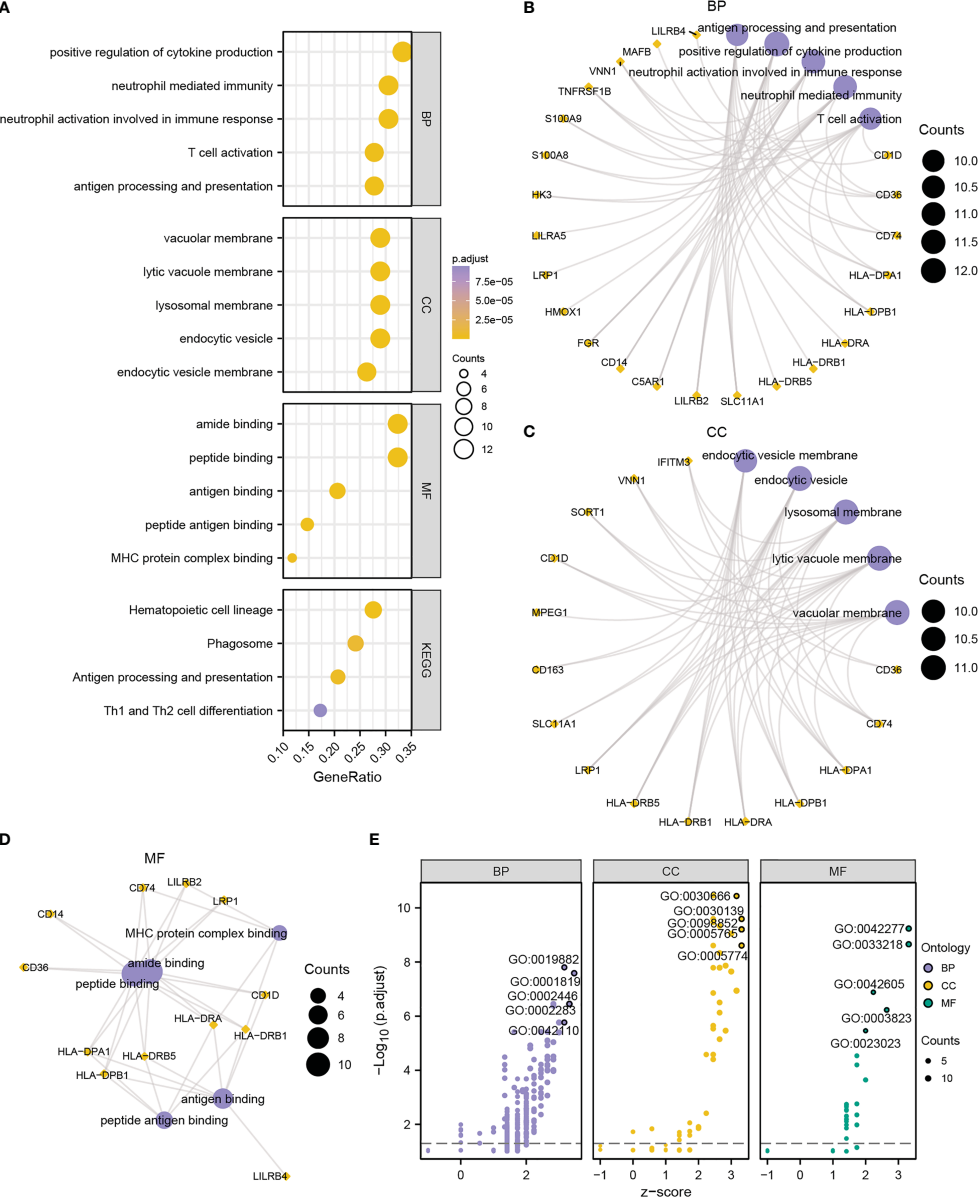
Functional annotation of DEGs between high- and low-risk groups. **(A)** Bubble chart showing the GO or KEGG analysis results. B-D. GO analysis for DEGs in BP **(B)**, CC **(C)**, and MF **(D)** terms. **(E)** Bubble chart displaying the GO analysis results of DEGs combined with logFC. The ordinate in the bubble chart **(A)** is the GO terms, and the length of the bubble from Y-axis stands for the GeneRatio value of GO terms. In network diagrams **(B–D)**, the orange dots represent specific genes, and the lavender circles represent specific pathways. In the bubble chart **(E)**, the lilac circles represent the BP pathway; the orange circles represent the CC pathway; and the green circles represent the MF pathway.

To explore the biological process between two risk groups, GO and KEGG analyses were conducted based on the 38 DEGs. GO analysis showed that the DEGs were mainly involved in immune responses, including positive regulation of cytokine, neutrophil mediated immunity, T cell activation and antigen processing and presentation (**Figure 8**, **Table S6**). The GO enrichment pathways of the 38 DEGs were mainly concentrated in the BP pathway (**Figure 8E**). KEGG analysis showed that the DEGs were mainly enriched in four pathways, hematopoietic cell lineage, phagosome, antigen processing and presentation, and Th1 and Th2 cell differentiation (**Figure 8A**, **Figure S5**, **Table S7**). These results indicated that 38 DEGs may play a crucial role in regulating AML TME.

To further explore the correlation between the 38 MMRGs’ prognosis-correlated DEGs and disease, DO analysis was performed. The DEGs were significantly (P-value< 0.05) enriched in parasitic protozoa infectious disease, multiple sclerosis, hypersensitivity reaction type IV disease, demyelinating disease, vasculitis, psoriatic arthritis, lung disease, sarcoidosis, parasitic infectious disease, and malaria (**Figure S6A**, **Table S8**). However, HMOX1 and HLA-DRB1 had the most correlations with diseases, and they were significantly correlated with seven diseases among the top ten diseases (**Figure S6B**).

To reveal the influence of MMRGs on AML occurrence, TCGA-LAML dataset was analyzed *via* GSEA to identify biological processes involved in two different groups. Surprisingly, these genes were remarkably associated with mitochondrial metabolism, drug resistance, and immunity, indicating that mitochondrial metabolism is crucial in immunoregulatory in TME and tumor drug resistance. Furthermore, genes highly in the high-risk group were significantly correlated with LSCs (**Figure S6D**), inflame pathway (**Figure S6E**), Ebola virus infection in host (**Figure S6F**), leukemia with MLL fusions (**Figure S6G**), mitochondrial translation (**Figure S6H**), glycolysis (**Figure S6I**), and multiple drug resistance (**Figure S6J**) (**Figures S6C–J**, **Table S9**) in the TCGA-LAML cohort.

### Identification of hub genes

**Figure 9:**
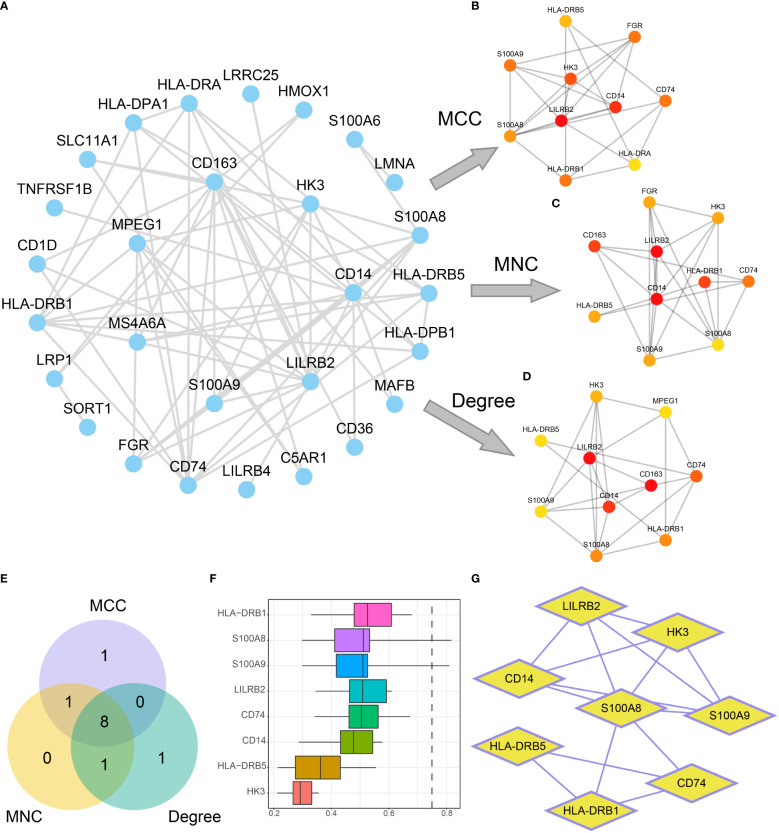
Construction of the PPI network. **(A)** PPI network of DEGs. **(B-D)**. Interaction networks of the top 10 DEGs in the PPI network obtained by the MCC **(B)**, MNC **(C)**, and Degree **(D)** algorithms. The color of the dots in the figure changes from yellow to red, indicating gradual increases in scores. **(E)** The Venn diagram of the top 10 DEGs obtained by the three algorithms. **(F, G)**. Functional similarity analysis results **(F)** and PPI network **(G)** of hub genes.

To further explore the molecular mechanisms underlying influence of mitochondrial metabolism on AML, A PPI network was built based on the 38 DEGs (**Figure 9A**). The top 10 DEGs were determined using Cytoscape’s cytoHubba plugin *via* MCC, MNC, and Degree algorithms (**Figures 9B–D**). Eight hub genes (CD14, CD74, HK3, HLA-DRB1, HLA-DRB5, LILRB2, S100A8, and S100A9) were identified with high connections (**Figure 9E**). Additionally, HLA-DRB1, S100A8 and S100A9 had the highest functional similarity with other hub genes (**Figures 9F, G**). The eight hub genes might play a pivotal role in immunoregulatory and treatment resistance.

### Characteristics of MMRGs in the TME immune regulation

**Figure 10:**
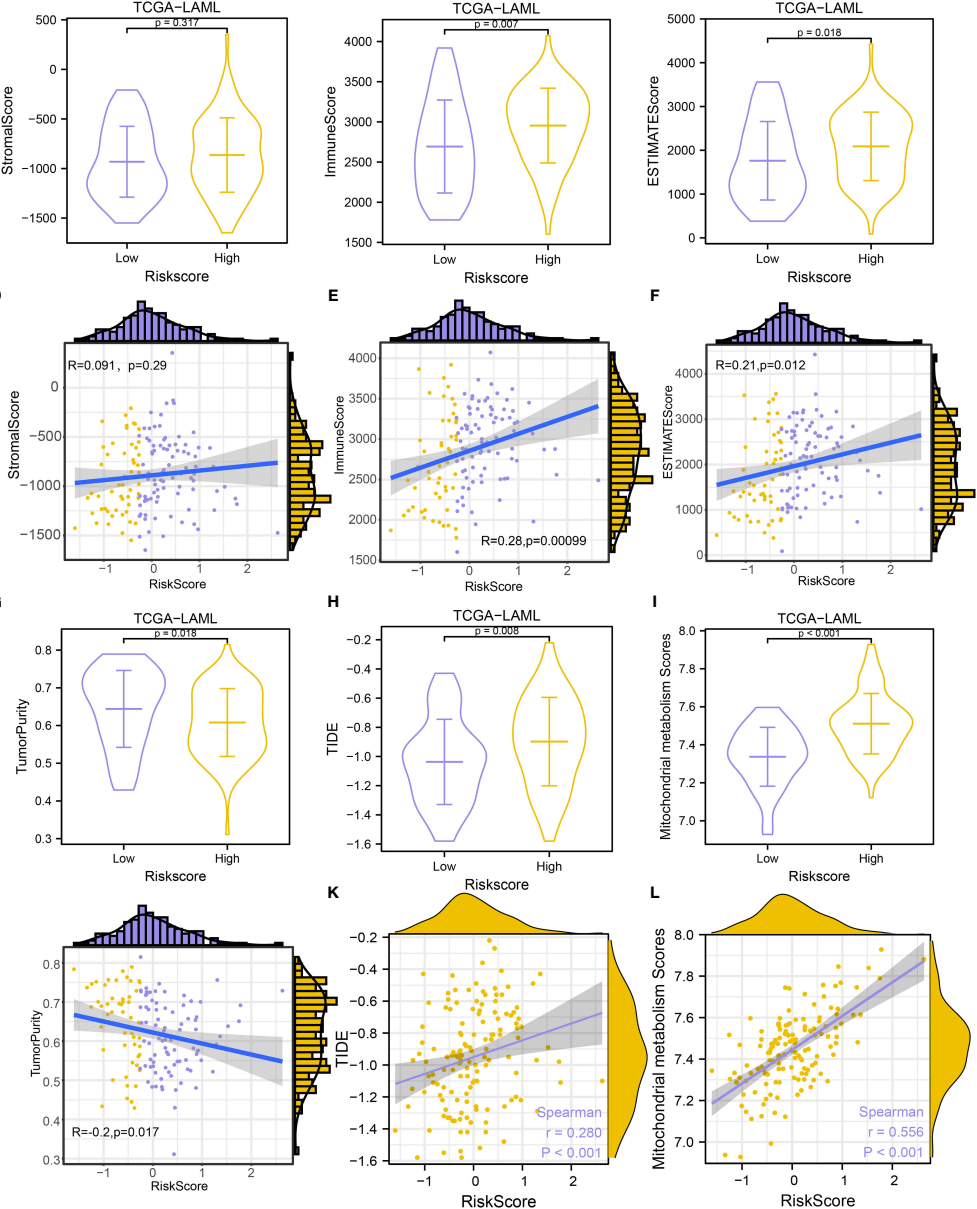
Immune infiltration estimation and correlation analysis. **(A-C)**. Comparison of StromaScore **(A)**, ImmuneScore **(B)**, and ESTIMATEScore **(C)** between groups. **(D-F)**. Scatterplots of the correlations between risk score and StromalScore **(D)**, ImmuneScore **(E)**, and ESTIMATEScore **(F)**. **(G-I)**. Comparison of Tumor Purity **(G)**, TIDE prediction score **(H)**, and MMs **(I)** between groups. **(J–L)**. Scatterplots of the correlations between risk score and Tumor Purity **(J)**, TIDE prediction score **(K)**, and MMs **(L)**.

**Figure 11:**
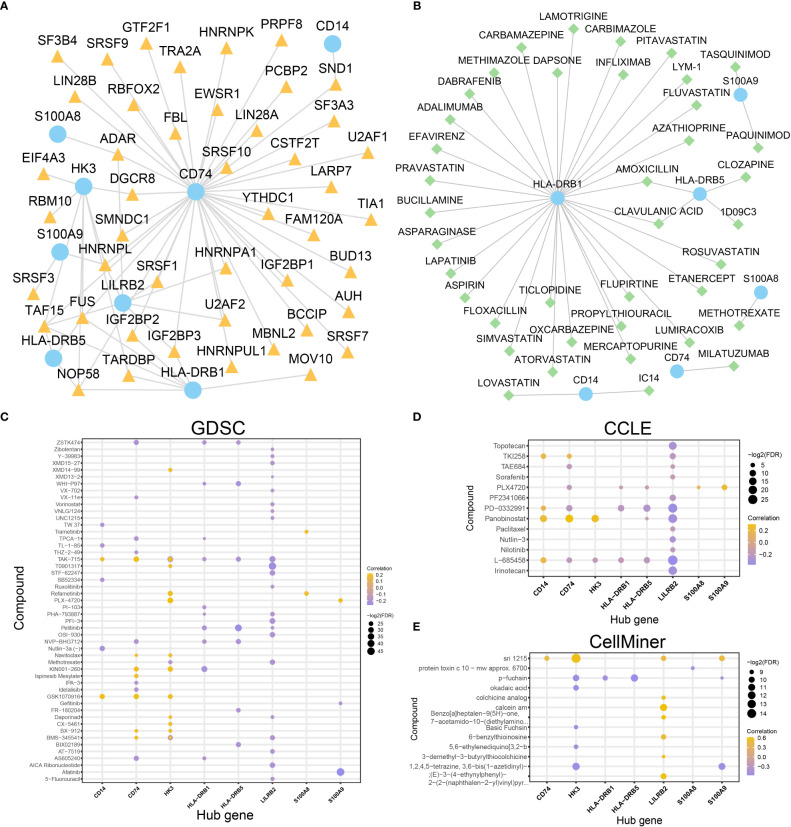
Construction of mRNA-RBP and mRNA-drugs interaction network and drug sensitivity analysis. **(A, B)**. mRNA-RBP **(A)** and mRNA-drugs **(B)** interaction networks for the identified hub genes. **(C–E)**. Drug sensitivity analysis results of hub genes in the GDSC database **(C)**, CCLE database **(D)**, and CellMiner database **(E)**. In the mRNA-RBP interaction network **(A)**, the blue circular block is mRNA; the orange triangle block is RBP. In the mRNA-drugs interaction network **(B)**, the blue circular block is mRNA; the light green diamond block is drug.

To further explore the influence of mitochondrial metabolism on TME, the ESTIMATE, CIBERSORT, and ssGSEA algorithms were employed to characterize the TME. Results showed that the stromal scores were comparable between the two groups (P-value > 0.05, **Figure 10A**). It was also observed that the high-risk group had higher immune scores (P-value = 0.007, **Figure 10B**), and higher ESTIMATE scores (P-value = 0.018, **Figure 10C**), but lower tumor purity compared with the low-risk group (P-value = 0.018, **Figure 10G**), suggesting that high-risk patients had high immune cell infiltration. Interestingly, we found that the risk score showed a positive nonsignificant association with the stromal score (R = 0.091, P-value = 0.29, **Figure 10D**), however, the risk score was significantly related to the immune score (R = 0.28, P-value = 0.00099, **Figure 10E**), ESTIMATE score (R = 0.21, P-value = 0.012, **Figure 11F**), and tumor purity (R = -0.2, P-value = 0.017, **Figure 10J**).

Although the high-risk group showed higher immune cell infiltration, M2 macrophages, gamma delta T cells, myeloid-derived suppressor cells (MDSCs) and regulatory T cells (Tregs) were significantly higher in the high-risk group (**Figures S7**, **S8**). Additionally, inhibitory receptors (IRs), such as CTLA4 and IL2RA, were significantly overexpressed in high-risk group patients (**Figure S9**). These indicated that patients in the high-risk group might have a strong immunosuppressive TME.

Given that immunotherapy has been considered as an important treatment for tumors, we also evaluate the immunotherapy response in AML patients in the TCGA-LAML dataset using the TIDE algorithm. The TIDE immunotherapy scores in high-risk patients were significantly higher compared with low-risk patients (P-value = 0.008, **Figure 10H**), suggesting that responses to immunotherapy might be higher in low-risk patients. Further analysis revealed that the risk scores were significantly related to TIDE immunotherapy scores (R = 0.280, P-value< 0.001, **Figure 10K**). High-risk patients showed significantly higher MMs (P-value< 0.001, **Figure 10I**), and risk scores were significantly positively related to MMs (R = 0.556, P-value< 0.001, **Figure 10L**).

To explore the role of hub genes in immune regulation, the correlations between immune infiltrating cells and hub genes were analyzed. By CIBERSORTx analysis, most immune cells were negatively correlated, with the highest positive correlation obtained between NK cells resting and Tregs in both groups, and the highest negative relationship was detected between monocytes and plasma cells (**Figures S7C-D**). Meanwhile, in both groups, 8 hub genes and 22 immune cells were found to be significantly correlated (P-value< 0.05) in both groups, and monocytes showed a significantly positive correlation with hub genes (**Figures S7D, E**). Through ssGSEA analysis, positive relationships were observed among the 15 immune cells in both groups (**Figures S8B, C**). Meanwhile, in the low-risk group of TCGA-LAML dataset, there were significant correlations (P< 0.05) between 15 immune cells and 8 hub genes, and most of them showed a significant positive correlation (**Figure S8D**). Specifically, CD14, HK3, LILRB2, S100A8, and S100A9 were significantly correlated with 15 immune cells. Similarly, significant positive correlations were found between 15 immune cells and 8 hub genes in high-risk group (**Figure S8E**), among which S100A8, S100A9, CD14, and LILRB2 were significantly correlated with 15 immune cells. Through correlation analysis, we found that the 8 hub genes were significantly positively correlated with the 11 immune-related genes (**Figure S9C**). These findings indicated that these hub genes were closely correlated with tumor immunity.

### Comparison of transcriptome and clinical traits between high- and low-risk groups

Given that tumor cells are characterized by genomic instability and altered metabolism, to determine the correlation between mitochondrial metabolism and DNA repair, we extracted 19 DNA repair genes from published literature. The analysis revealed that ERCC1, FANCC, FEN1, MGMT, MLH1, RAD23A, and XPC were upregulated in high-risk patients whereas MBD4 and WRN were downregulated in low-risk patients (**Figure S9B**). As displayed in **Figure S9D**, 8 hub genes were significantly related to 19 DNA repair genes, with WRN showing a negative correlation with the 8 hub genes.

To better illustrate the characteristic differences between the two groups, we compared clinical traits of the two groups. The proportion of patients under 60 years old in the low-risk group was significantly higher than that of high-risk group (**Figure S9E**), while there was no difference in sex between the high- and low-risk groups (**Figure S9F**). The proportion of OS events in low-risk group was markedly lower than that in the high-risk group (**Figure S9G**).

### Identification of potential druggable targets

To investigate the regulatory mechanism of hub genes in AML and whether all of the hub genes are druggable targets, mRNA-RBP interaction analysis and mRNA-drug interaction analysis were conducted. An mRNA-RBP network with eight hub genes and 45 RBPs (**Figure 11A**) was constructed (69 pairs of mRNA-RBP interaction relationships) (**Table S10**). Meanwhile, 39 potential drugs targeting six hub genes (CD14, CD74, HLA-DRB1, HLA-DRB5, S100A8, S100A9) were identified (**Figure 11B**, **Table S11**). Besides, 31 drugs or molecular compounds targeting the HLA-DRB1 gene were identified.

What’s more, the drug sensitivity was predicted based on the mRNA expression profile and drug activity data of the eight hub genes in the GDSC, CCLE, and CellMiner database *via* pRRophetic algorithm. A total of 50 and 13 drugs interacted with eight hub genes in the GDSC and CCLE databases, respectively (**Figures 11C, D**). Fourteen drugs interacted with the other seven hub genes (except for the CD14 gene) in the CellMiner database (**Figure 11E**). These results indicate that the eight hub genes were all druggable targets. The majority of the hub genes are related to immune regulation, however small molecular inhibitors, such as BCL2 inhibitors, might target these genes. This suggested that treatment targeting mitochondrial metabolism might improve the TME of AML patients.

### Prognostic performance of MMRGs prognosis model

**Figure 12:**
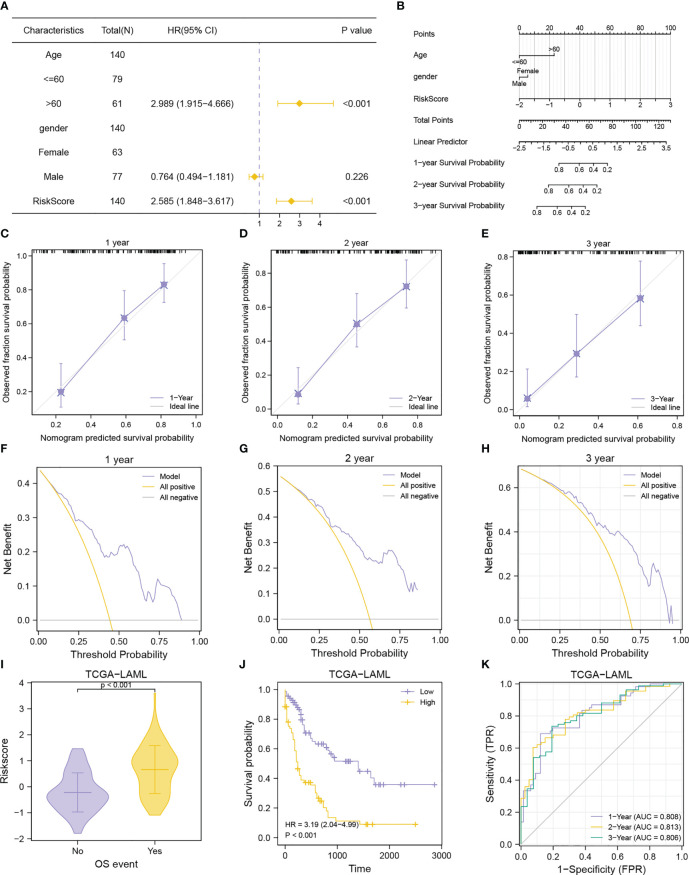
Prognostic performance of the MMRGs prognostic model. **(A, B)**. Forest plot **(A)**, nomogram **(B)** of multivariate Cox regression model with risk score and clinical variable. **(C-E)**. Calibration curves of multivariate Cox regression model nomogram for the 1-year **(C)**, 2-year **(D)**, and 3-year **(E)** outcomes. F-H. DCA plots of multivariate Cox regression model for the 1-year **(F)**, 2-year **(G)**, and 3-year **(H)** outcomes. **(I-K)**. Comparison of OS events **(I)**, prognostic KM curve **(J)**, and time-dependent ROC curve **(K)** of the risk score + age + gender prognosis model.

To evaluate the performance of the MMRGs prognosis model, univariate and multivariate Cox regression were conducted based on risk scores combined with 2 independent clinical variables (age, and gender). The results are shown in the forest plot (**Figure 12A**). Results indicated that the risk score remained an independent predictor of survival in the multivariate Cox regression analysis (P-value< 0.001, **Table 3**). A nomogram was developed which revealed that the risk score contributed the most risk points compared with other clinical variables (**Figure 12B**). Additionally, we conducted calibration analysis to evaluate the correctness of the model. The results indicated that the model could accurately predict AML patients’ OS at 1-, 2-, and 3-year (**Figures 12C–E**). The clinical benefit of this prognosis model at 1-, 2- and 3-year was also determined using the DCA (**Figures 12F–H**). This model displayed many clinical benefits over time.

**Table 3:** Cox regression to MMRGs prognosis model and identify clinical features associated with OS in TCGA-LAML.

Characteristics	Total(N)	Univariate analysis		Multivariate analysis	
		Hazard ratio (95% CI)	P value	Hazard ratio (95% CI)	P value
Age	140				
<=60	79	Reference			
>60	61	3.333 (2.164-5.134)	<0.001	2.989 (1.915-4.666)	<0.001
gender	140				
Female	63	Reference			
Male	77	1.030 (0.674-1.572)	0.892	0.764 (0.494-1.181)	0.226
RiskScore	140	2.718 (1.972-3.747)	<0.001	2.585 (1.848-3.617)	<0.001

The AML patients in the TCGA-LAML cohorts were grouped according to the OS to explore differences in risk scores between different OS event groups. Patients in the death group had higher risk scores (**Figures 12I**). Patients with lower risk scores had longer survival as determined from the KM curves (**Figure 12J**). Data shown in **Figures 12K** revealed that the prognosis model based on MMRGs risk score had high predictive accuracy (AUC1 = 0.808, AUC2 = 0.813, AUC3 = 0.806).

## Discussion

AML is a common hematologic malignancy characterized by poor prognosis and high recurrence rates. The 5-year survival rate of AML is low because of disease relapse and lack of precise treatment for each patient. Therefore, assessing the resistance mechanism and finding novel potential molecular targets for AML therapeutic intervention is essential. Genomic aberrations and functional heterogeneity have been studied to understand AML heterogeneity (59, 60). Although most somatic mutations responsible for AML have been identified, the lack of targeted therapies for numerous mutations limits the widespread clinical success of mutation-specific treatments. Some studies have shown that mitochondrial metabolism promotes AML tumorigenesis, progression, and treatment resistance (15, 61). Therefore, mitochondrial metabolism can be a potential therapeutic target in AML. In this study, a prognosis model including five MMRGs that can accurately predict outcomes of AML patients was constructed. Additionally, we identified different immune infiltration status and immunotherapeutic responses between high- and low- risk groups. Potential drugs were further identified.

In this study, overall somatic mutation frequency of 31 MMRGs was extremely low in AML, which was consistent with a recent study (62). Noteworthy, LSC transcriptional and epigenetic signatures are mainly independent of genetic mutations (63). Moreover, LSC protein expression profiles are enriched for biomarkers of OXPHOS and LSC dependent on mitochondrial metabolism for survival (64). Next, MMs based on the expression of 31 MMRGs were calculated, which were notably associated with the OS and FAB stages of AML patients in all cohorts. By WGCNA analysis and univariate Cox regression analysis, we identified 9 prognosis-related MMRGs (POLG, NDUFV1, BCS1L, TACO1, SUCLG1, SDHA, NDUFA1, NDUFS2 and ECHS1). POLG, the sole polymerase responsible for mitochondrial DNA replication, have been reported to be involved in the proliferation and differentiation of AML cells *in vitro* and *in vivo (* 65). NDUFV1, subunits of ETC complex I, was demonstrated to be overexpressed in AML LSCs (66). BCS1L encodes a mitochondrial protein that functions as a chaperone in the assembly of respiratory chain complex III. A recent study revealed that knockdown of BCS1L could reduce AML proliferation and oxidative metabolism (67). TACO1, encoding a translational activator of cytochrome c oxidase 1, the function of which is poorly understood (68). Previous studies reported that SUCLG1 and SDHA are independent prognosis predictors in AML (69–71). SUCLG1 participate in regulate ETC, regulate TCA cycle activities, and regulate mitochondrial respiration-dependent energy production. SDHA is a component of ETC complex II, that promotes oxidative cell phosphorylation and ATP production through glutathionylation, which is required for the survival of AML LSCs (71). Venetoclax + azacytidine treatment can inhibit SDHA glutathionylation and target LSCs in AML patients (72). NDUFA1, subunit of mitochondrial complex I, was associated with the prognosis of head and neck squamous cell carcinoma (73). ECHS1 plays an important role in phospholipid metabolism, tumor occurrence, development, and drug resistance (74). NDUFS2 is involved in cell growth, metabolism, apoptosis, and necrosis. A recent study reported that antitumor compound SMIP004-7 could eradicate drug-resistant cancer cells and promote anticancer immune surveillance by targeting NDUFS2 (75). These findings confirmed that mitochondrial metabolism plays a crucial role in tumor development and progression.

The 9 MMRGs were further used for Lasso regression, and 5 MMRGs were selected (SUCLG1, SDHA, NDUFA1, ECHS1 and NDUFS2). Subsequently, a novel AML risk signature combining the 5 MMRGs was developed *via* integrative analysis of TCGA and GEO databases. The low-risk group showed a more favorable prognosis in both training and validation cohorts. The signature could accurately predict the prognosis of AML patients at 1-, 2- and 3-year, showing a stable strong predictive power. We then combined risk scores and clinical characteristics (age and gender) to develop a nomogram, indicating that risk score was an independent predictor. The predictive capability of our risk signature is equivalent to that of a signature including four genes (23) and a signature including six genes (22). However, our signature can better predict antitumor immune responses and promotes the finding of biological targets for immune treatments. This is the first study to assess the prognostic value of ECHS1, NDUFA1, and NDUFS2 expression on the overall survival of AML patients. What’s more, we found that AML BM samples had significantly higher ECHS1 and NDUFS2 expression than normal BM samples by IHC assay. According to these findings, we speculate that ECHS1 and NDUFS2 may be essential regulators in influencing the prognosis of AML patients.

The DEGs between high- and low-risk groups were mainly related to immune regulation and mitochondrial metabolism, such as positive regulation of cytokine production, neutrophil activation, T cell activation, and Th1 and Th2 cell differentiation. This indicated that these two groups were affected by different immune regulation. Increasing studies have demonstrated that the leukemic microenvironment is involved in outcomes and treatment responses of AML patients (76, 77). By immune infiltration analysis, we found that the high-risk group showed higher immune scores, but increased ratio of gamma delta T cells, M2 macrophages, MDSCs and Tregs and increased expression of CTLA4 and IL2RA. In many cancers, immunoregulatory mechanisms have been proved to hamper antitumor immunotherapy, including ligand-mediated engagement of IRs on effector cells, such as CTLA4, and induction of immunosuppressive cell subsets, such as Tregs or MDSCs (78–80). IL2RA was demonstrated to be involved in inferior outcomes of AML patients (81). Several clinical trials have assessed immunotherapy for AML, including checkpoint inhibitors, CAR T cells, multispecific antibodies, and vaccines (82, 83). Nonetheless, immunotherapy has only benefited a few patients. Therefore, effective biomarkers are needed to predict the immunotherapy response of AML patients. Herein, the high-risk group showed more likelihood to experience immune escape or immune dysfunction, indicating poor response to immunotherapy. This result suggests that mitochondrial metabolism-related genes may be a new therapeutic target.

Drug repurposing, the new use of old drugs, can accelerate drug development and reduce the cost (84). Hub genes identified from 38 DEGs between high- and low-risk groups were used to explore potential drugs by protein-drug interaction analysis and drug sensitivity analysis with public databases. 77 ideal compounds for targeting hub genes were identified (PI3K inhibitor, BCL-2 inhibitor, CDK inhibitor, MDM2 inhibitor, tyrosine kinase inhibitor, etc.). Tasquinimod, an inhibitor of S100A9, is currently in a phase Ib/IIa clinical trial in multiple myeloma patients (NCT04405167). A recent study demonstrated that Tasquinimod could inhibit the MDSC suppressive ability and promote T cell activation in myeloma BM microenvironment (85). As we showed, the TME of the high-risk group were immune suppressive with higher MDSCs and Tregs infiltration and poor immunotherapy response. Additionally, Tasquinimod was reported to target HDAC4 to inhibit tumor proliferation (86). Increasing studies have shown histone deacetylases play pivotal roles in leukemogenesis and stemness maintenance of AML (87, 88). These indicates that Tasquinimod might be a candidate drug for AML. Navitoclax, a BCL-2 inhibitor, have been demonstrated as a single agent or in combine with other drugs to successfully eradicate leukemia cells (89, 90). Venetoclax, another BCL-2 inhibitor, can reduce mitochondrial respiration, induce TCA cycle inhibition, and activate reductive carboxylation in AML cells, thus inducing apoptosis (91–93). AXL inhibitor, a tyrosine kinase inhibitor, combined with venetoclax, can eradicate AML primitive cells by perturbating the process of OXPHOS (94). These findings indicated that these drugs might have effects on AML. In this study, GSEA showed that genes associated with multiple drug resistance were overexpressed in the high-risk group, indicating that mitochondrial metabolism is related to the resistance treatment in AML. Similarly, other studies reported that OXPHOS and mitochondrial metabolism can promote chemotherapy resistance in AML patients (95), suggesting that mitochondrial metabolism is a promising therapy target for AML. In addition, some studies have demonstrated that OXPHOS inhibitors could effectively eradicate leukemia cells and are being evaluated in clinical trials, such as IACS-010759 (96), Mubritinib (61), Ammocidin (97), devimistat (98) and ONC212 (99). Although drug repurposing is an effective strategy to search for therapeutic candidates, but the anti-tumor mechanism of these drugs still needs further investigation.

However, this study has some limitations. First, there was incomplete data for all variables of AML patients, and thus the MMRGs risk score + age + gender prognosis model was not validated with the GSE12417 and GSE37642 datasets. Although 736 AML patients from three datasets were included, the prediction value of five MMRGs was evaluated using only 314 AML patients. Therefore, more independent AML cohorts and prospective studies are needed to validate the prognostic signature. Second, this study was analyzed *via* bioinformatics based on public datasets only. Sufficient clinical samples and corresponding clinical data are needed to verify the prediction value of these MMRGs. Third, it is unclear how these MMRGs work together to affect immune infiltration, immunotherapy response, and drug sensitivity in AML patients. Analyses of the data were based solely on bioinformatics and IHC assays in this study. Therefore, *in vitro* and *in vivo* experiments are needed to further reveal these mechanisms.

## Conclusions

Taken together, a novel five MMRGs signature that could accurately predict the prognosis outcomes and immune status in AML patients was successfully developed and validated. This study may provide novel insights into predicting clinical outcomes for AML patients. Meanwhile, this study also develops theoretical guidelines for improving immunotherapy and personalized antitumor treatment.

## Data availability statement

The datasets presented in this study can be found in online repositories. The names of the repository/repositories and accession number(s) can be found in the article/**Supplementary material**.

## Ethics statement

This study involving human samples was approved by the Ethics Committee of Zhongnan Hospital of Wuhan University (No.2020010). The patients/participants provided their written informed consent to participate in this study.

## Author contributions

XT and FZ designed the study. XT acquired and analyzed the data. XT wrote the manuscript. FZ revised the manuscript. All authors contributed to the article and approved the submitted version.

## Supplementary material

Supplementary Figure 1 Normalization of the AML datasets. A-B. Boxplots of the TCGA-LAML dataset before **(A)**, and after **(B)** normalization. **(C, D)** Boxplots of the GSE12417 dataset before **(C)**, and after **(D)** normalization. **(E, F)** Boxplots of the GSE37642 dataset before **(E)**, and after **(F)** normalization.

Supplementary Figure 2 Mutation analysis of MMRGs and correlation of MMs and FAB stages in TCGA-LAML cohort. **(A)** SNP of MMRGs in AML patients. **(B)** Comparisons of MMs among different AML FAB subgroups. *P-value< 0.05, **P-value< 0.01, ***P-value< 0.001.

Supplementary Figure 3 Correlation analysis for the module MMRGs. A. Heatmap showing the correlation among module MMRGs in the TCGA-LAML dataset. **(B-F)** Scatter plots for the correlations between BCS1L and NDUFV1 **(B)**; MPV17 and NDUFV1 **(C)**; DGUOK and NDUFA1 **(D)**; NDUFA13 and NDUFS7 **(E)**; NDUFS7 and NDUFV1 **(F)**.

Supplementary Figure 4 Differential expression of 17 module MMRGs in the two AML clusters. **(A)** The expression of 17 module MMRGs in the two AML clusters. **(B-R)** ROC curves for discriminating the two AML subtypes from the TCGA-LAML cohort on BCS1L **(B)**, COX10 **(C)**, DGUOK **(D)**, ECHS1 **(E)**, ETHE1 **(F)**, MPV17 **(G)**, NDUFA1 **(H)**, NDUFA13 **(I)**, NDUFS2 **(J)**, NDUFS7 **(K)**, NDUFV1 **(L)**, POLG **(M)**, SDHA **(N)**, SUCLG1 **(O)**, SURF1 **(P)**, TACO1 **(Q)**, TIMM8A **(R)**. **P-value < 0.01, ***P-value < 0.001.

Supplementary Figure 5 KEGG pathway map of DEGs between high- and low-risk groups. **(A-D)** KEGG pathway maps of hematopoietic cell lineage **(A)**, phagosome **(B)**, antigen processing and presentation **(C)**, Th1 and Th2 cell differentiation **(D)** enriched by KEGG analysis based on the DEGs between high- and low-risk groups.

Supplementary Figure 6 DO analysis of DEGs and GSEA analysis. **(A, B)** Histogram **(A)** and network diagram **(B)** displaying DO analysis results of the DEGs. C. Seven main biological characteristics of GSEA analysis. D-J. Genes in the TCGA-LAML dataset were significantly enriched in leukemic stem cell **(D)**, inflame pathway **(E)**, Ebola virus infection in host **(F)**, leukemia with MLL fusion **(G)**, mitochondrial translation **(H)**, glycolysis **(I)**, and multiple drug resistance **(J)** pathways.

Supplementary Figure 7 CIBERSORTx immune infiltration analysis of high- and low-risk groups. **(A)** Immune cell infiltration in high- and low-risk groups by CIBERSORTx analysis. **(B, C)** Correlations between each immunocyte infiltration level in the low-risk group **(B)** and high-risk group **(C)**. **(D, E)** Heatmap showing the correlations between hub genes and immunocyte infiltration level in low-risk group **(D)** and high-risk group **(E)**.

Supplementary Figure 8 ssGSEA analysis for high- and low-risk groups. **(A)** Immune cell infiltration in high- and low-risk groups as determined by ssGSEA analysis. **(B, C)**. Correlations between each immunocyte infiltration level in low- **(B)** and high-risk group **(C)**. **(D, E)**. Heatmap displaying the correlations between hub genes and immunocyte infiltration level in low- **(D)** and high-risk group **(E)**. *P-value< 0.05, **P-value< 0.01, ***P-value< 0.001, ns, no significance.

Supplementary Figure 9 Difference analysis between high- and low-risk groups. **A, B**. Expression of immune-related genes **(A)** and DNA repair genes **(B)** in the AML patients. **C, D**. Relationships between hub genes and immune-related genes **(C)** and DNA repair genes **(D)**. **E-G**. Stacked histogram of the proportions of age **(E)**, gender **(F)**, OS event **(G)**. *P-value< 0.05, **P-value< 0.01, ***P-value< 0.001.
