# Supplementary figures and images for: Integrated bioinformatic analysis of mitochondrial metabolism-related genes in acute myeloid leukemia
Source: Front Immunol. 2023 Apr 17;14:1120670. doi: 10.3389/fimmu.2023.1120670 (PMC10149950; doi:10.3389/fimmu.2023.1120670)

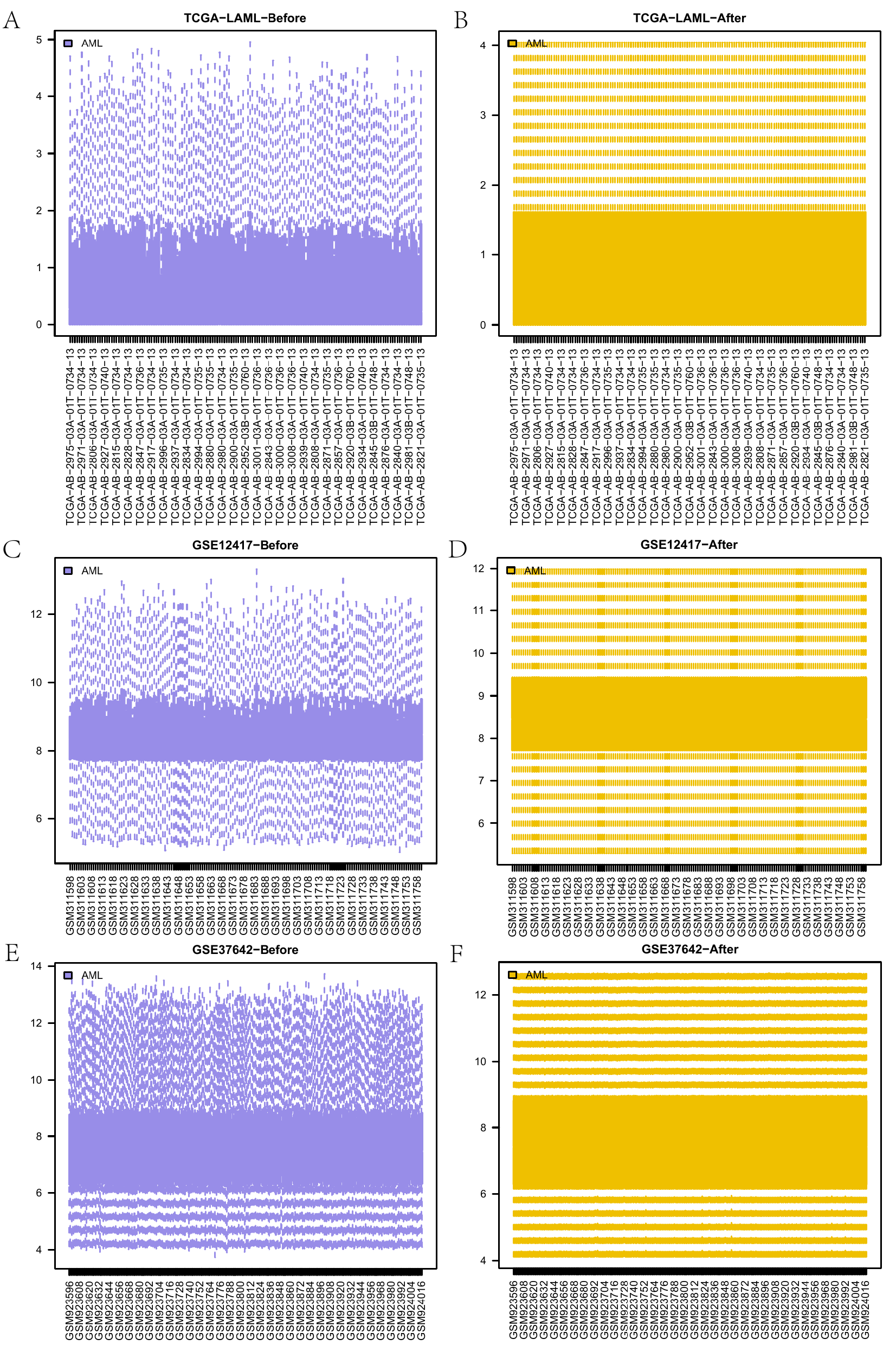

Supplement: Supplementary Figure 1 — Normalization of the AML datasets. A-B. Boxplots of the TCGA-LAML dataset before (A), and after (B) normalization. (C, D) Boxplots of the GSE12417 dataset before (C), and after (D) normalization. (E, F) Boxplots of the GSE37642 dataset before (E), and after (F) normalization. [file Image_1.tiff]

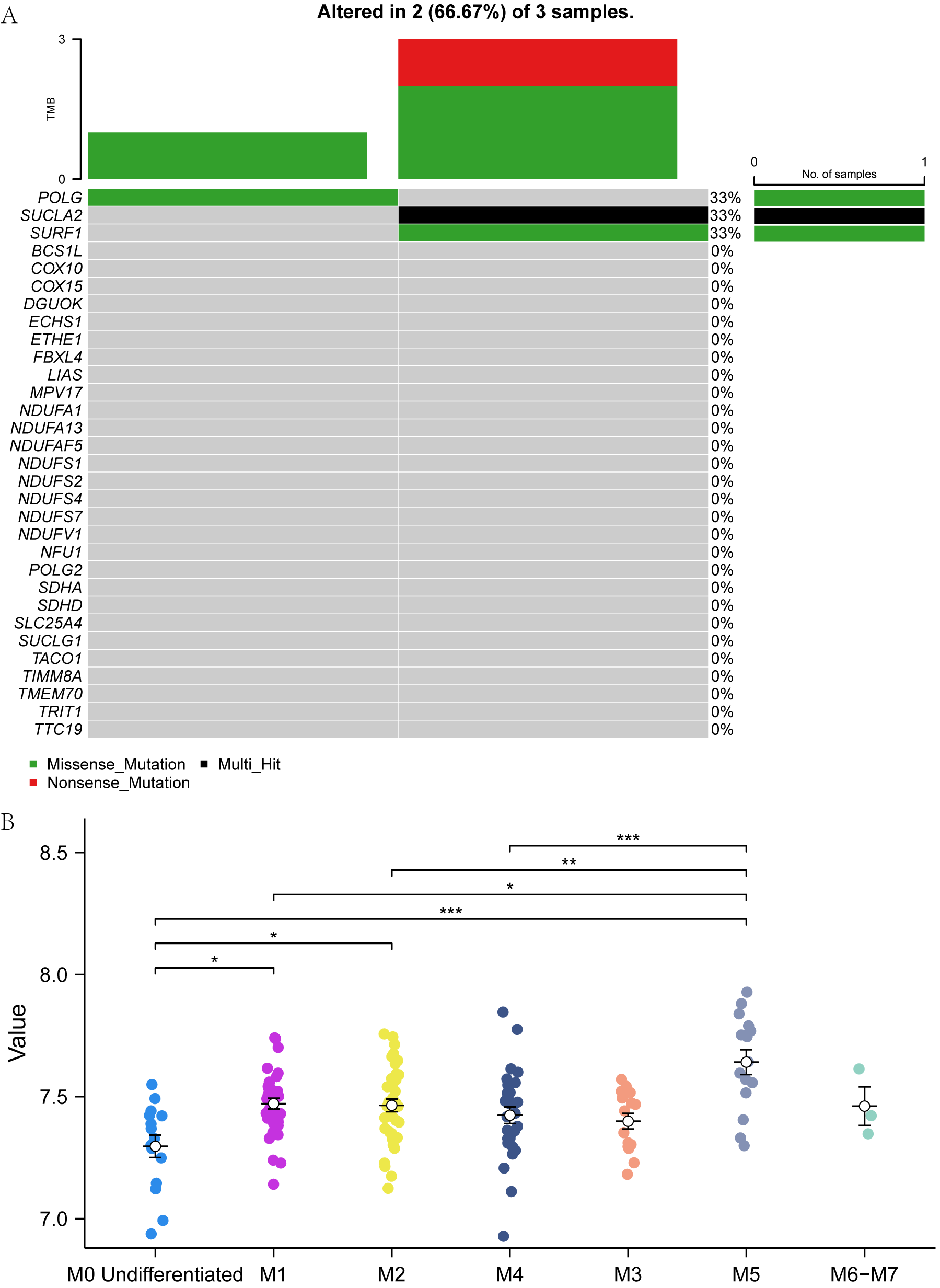

Supplement: Supplementary Figure 2 — Mutation analysis of MMRGs and correlation of MMs and FAB stages in TCGA-LAML cohort. (A) SNP of MMRGs in AML patients. (B) Comparisons of MMs among different AML FAB subgroups. *P-value< 0.05, **P-value< 0.01, ***P-value< 0.001. [file Image_2.tif]

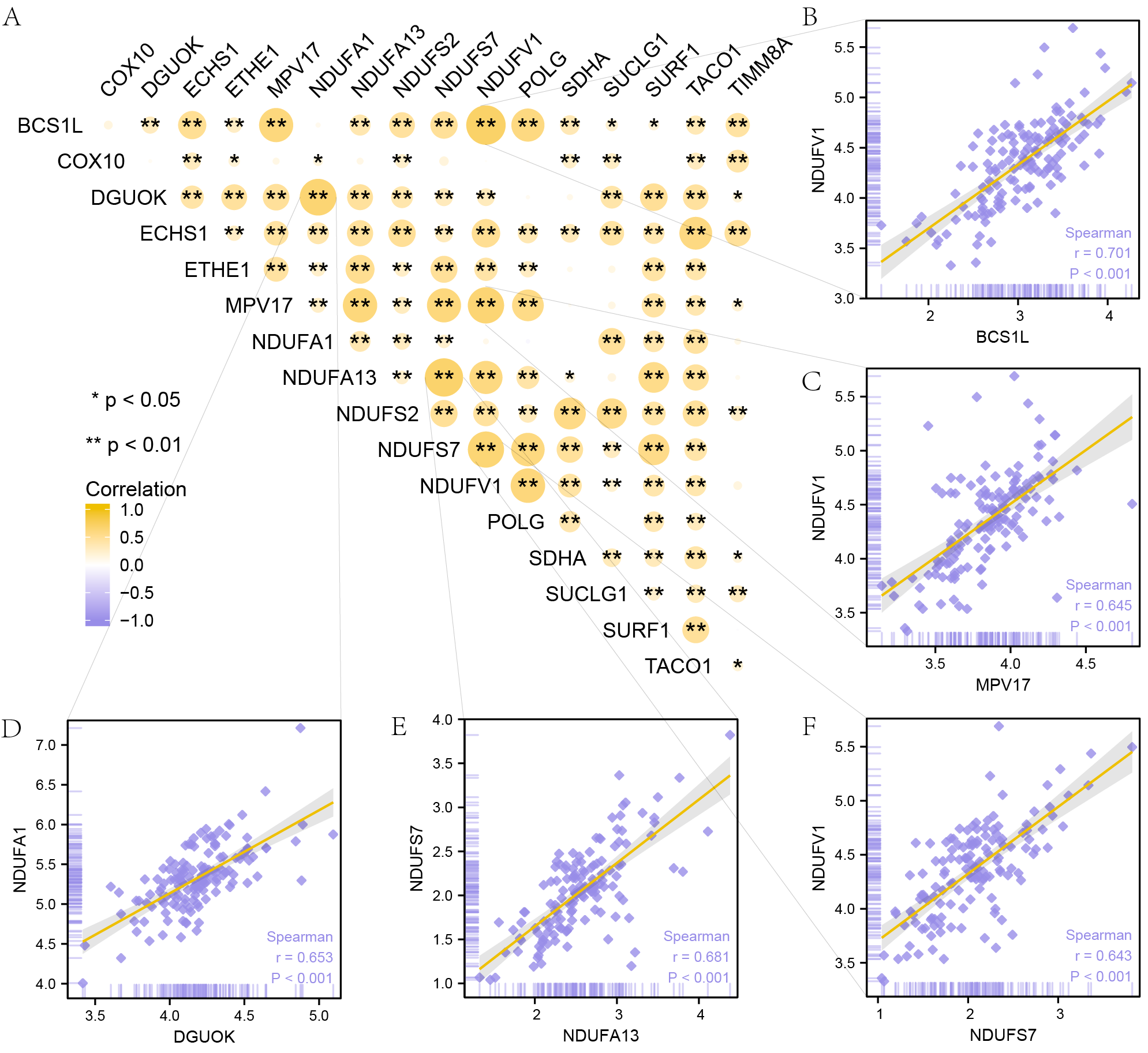

Supplement: Supplementary Figure 3 — Correlation analysis for the module MMRGs. A. Heatmap showing the correlation among module MMRGs in the TCGA-LAML dataset. (B-F) Scatter plots for the correlations between BCS1L and NDUFV1 (B); MPV17 and NDUFV1 (C); DGUOK and NDUFA1 (D); NDUFA13 and NDUFS7 (E); NDUFS7 and NDUFV1 (F). [file Image_3.tif]

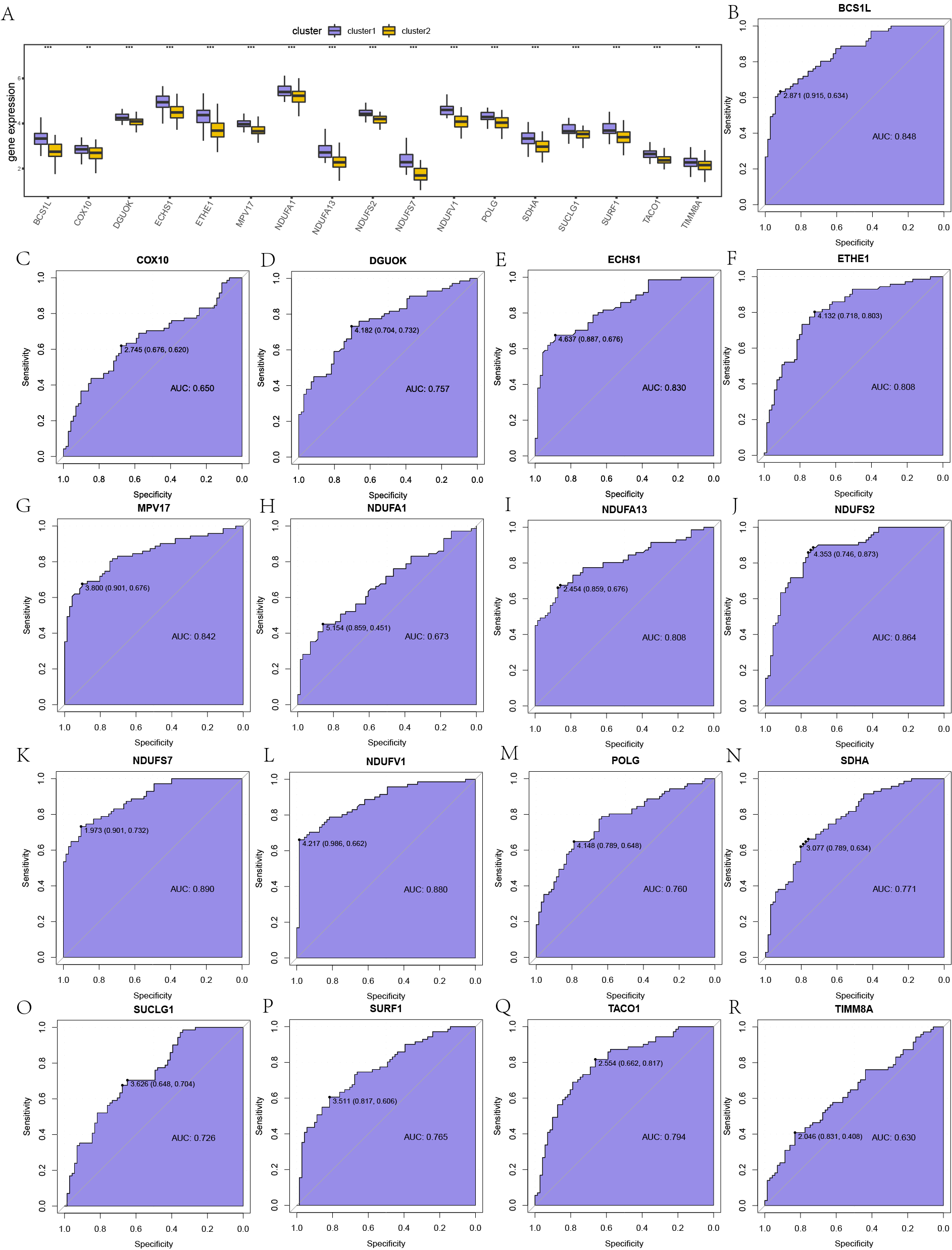

Supplement: Supplementary Figure 4 — Differential expression of 17 module MMRGs in the two AML clusters. (A) The expression of 17 module MMRGs in the two AML clusters. (B-R) ROC curves for discriminating the two AML subtypes from the TCGA-LAML cohort on BCS1L (B), COX10 (C), DGUOK (D), ECHS1 (E), ETHE1 (F), MPV17 (G), NDUFA1 (H), NDUFA13 (I), NDUFS2 (J), NDUFS7 (K), NDUFV1 (L), POLG (M), SDHA (N), SUCLG1 (O), SURF1 (P), TACO1 (Q), TIMM8A (R). **P-value < 0.01, ***P-value < 0.001. [file Image_4.tif]

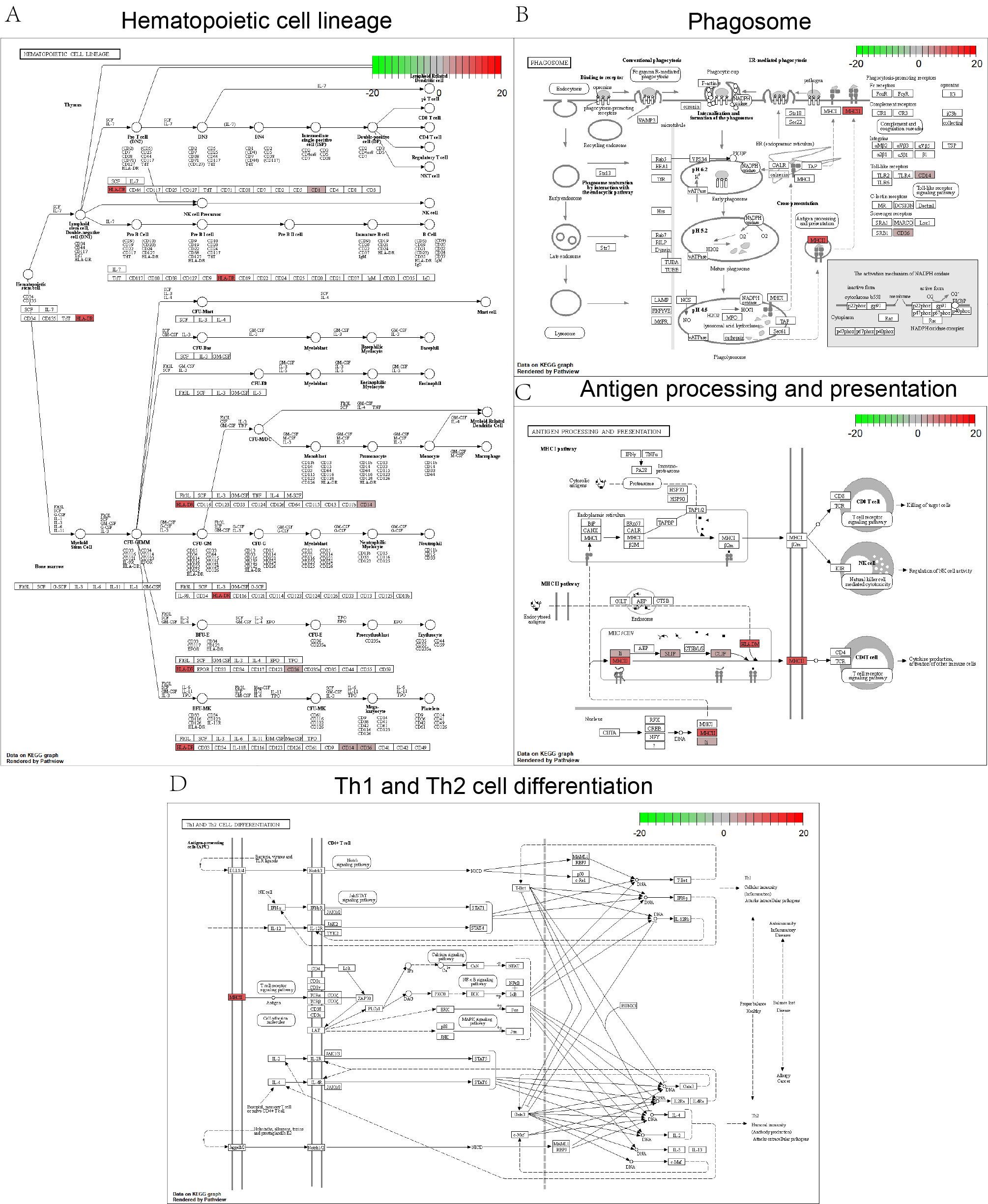

Supplement: Supplementary Figure 5 — KEGG pathway map of DEGs between high- and low-risk groups. (A-D) KEGG pathway maps of hematopoietic cell lineage (A), phagosome (B), antigen processing and presentation (C), Th1 and Th2 cell differentiation (D) enriched by KEGG analysis based on the DEGs between high- and low-risk groups. [file Image_5.tif]

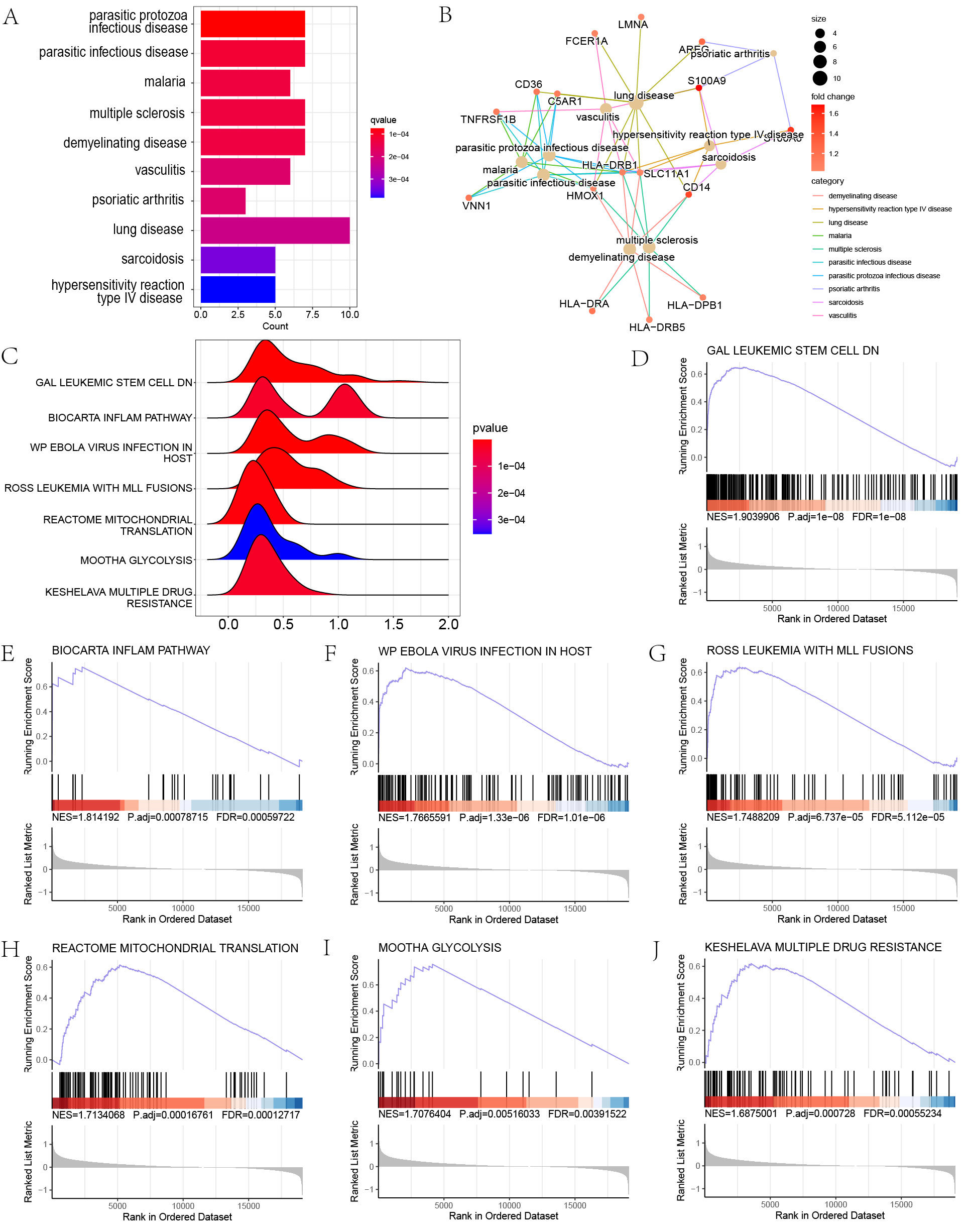

Supplement: Supplementary Figure 6 — DO analysis of DEGs and GSEA analysis. (A, B) Histogram (A) and network diagram (B) displaying DO analysis results of the DEGs. C. Seven main biological characteristics of GSEA analysis. D-J. Genes in the TCGA-LAML dataset were significantly enriched in leukemic stem cell (D), inflame pathway (E), Ebola virus infection in host (F), leukemia with MLL fusion (G), mitochondrial translation (H), glycolysis (I), and multiple drug resistance (J) pathways. [file Image_6.tif]

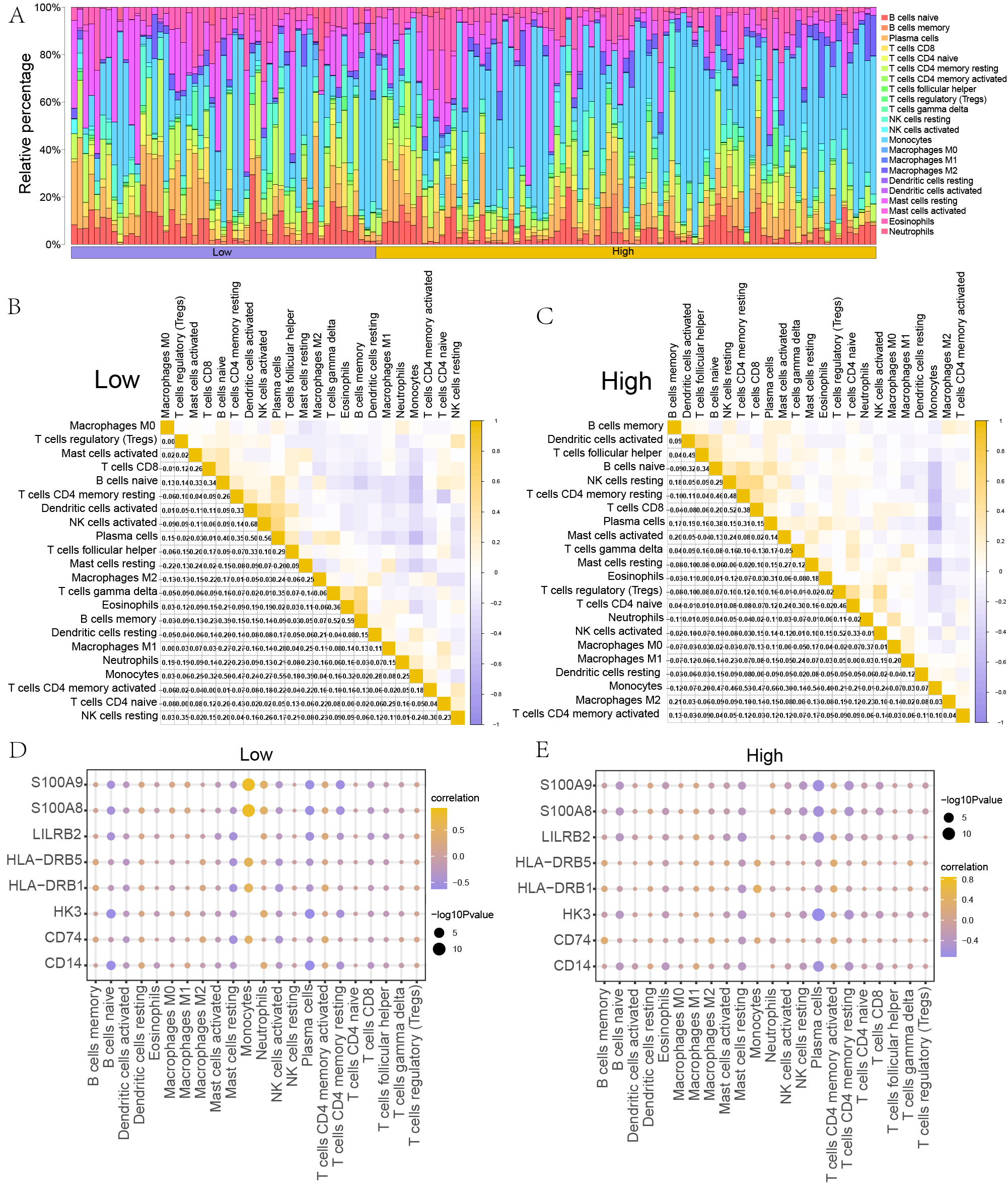

Supplement: Supplementary Figure 7 — CIBERSORTx immune infiltration analysis of high- and low-risk groups. (A) Immune cell infiltration in high- and low-risk groups by CIBERSORTx analysis. (B, C) Correlations between each immunocyte infiltration level in the low-risk group (B) and high-risk group (C). (D, E) Heatmap showing the correlations between hub genes and immunocyte infiltration level in low-risk group (D) and high-risk group (E). [file Image_7.tif]

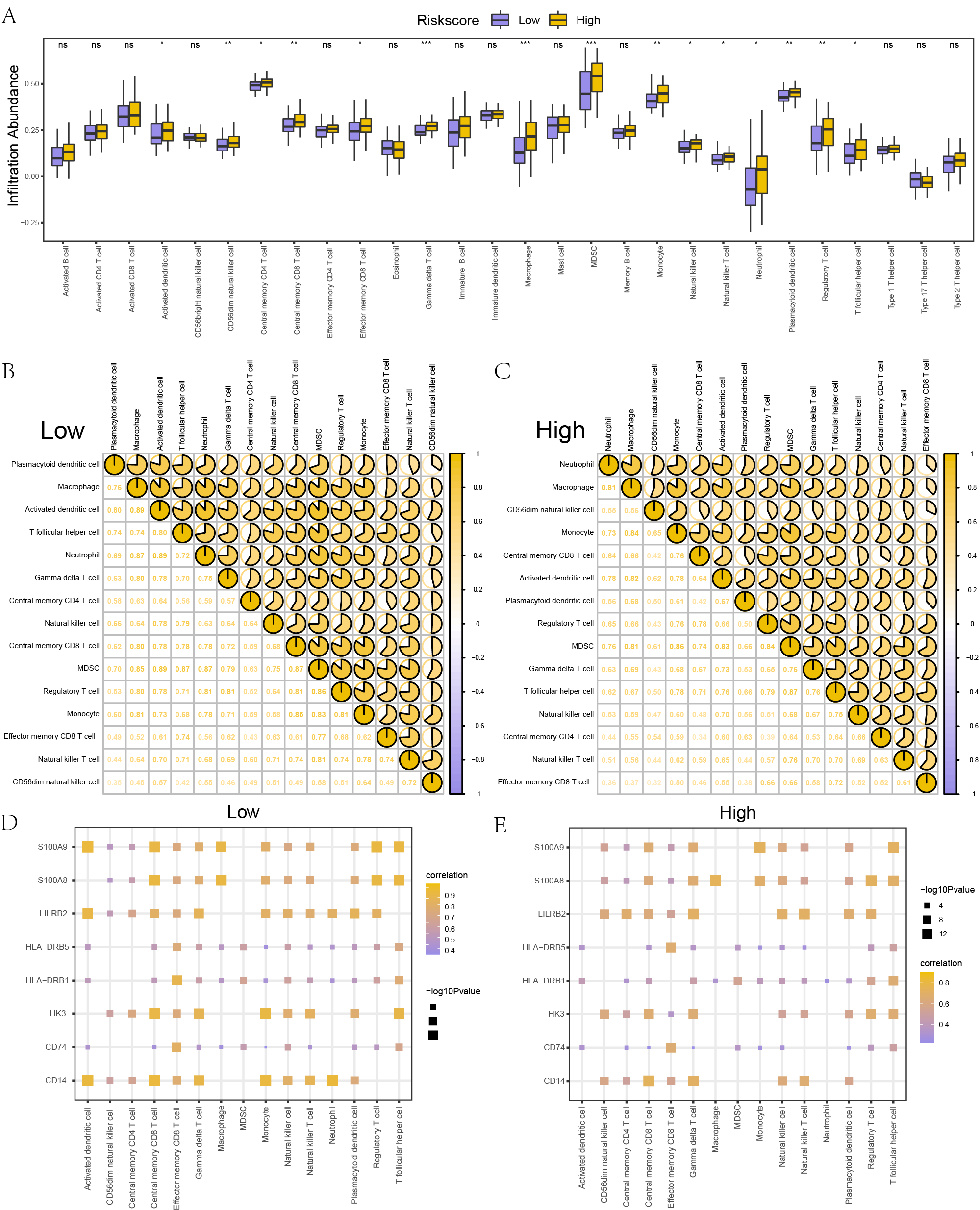

Supplement: Supplementary Figure 8 — ssGSEA analysis for high- and low-risk groups. (A) Immune cell infiltration in high- and low-risk groups as determined by ssGSEA analysis. (B, C). Correlations between each immunocyte infiltration level in low- (B) and high-risk group (C). (D, E). Heatmap displaying the correlations between hub genes and immunocyte infiltration level in low- (D) and high-risk group (E). *P-value< 0.05, **P-value< 0.01, ***P-value< 0.001, ns, no significance. [file Image_8.tif]

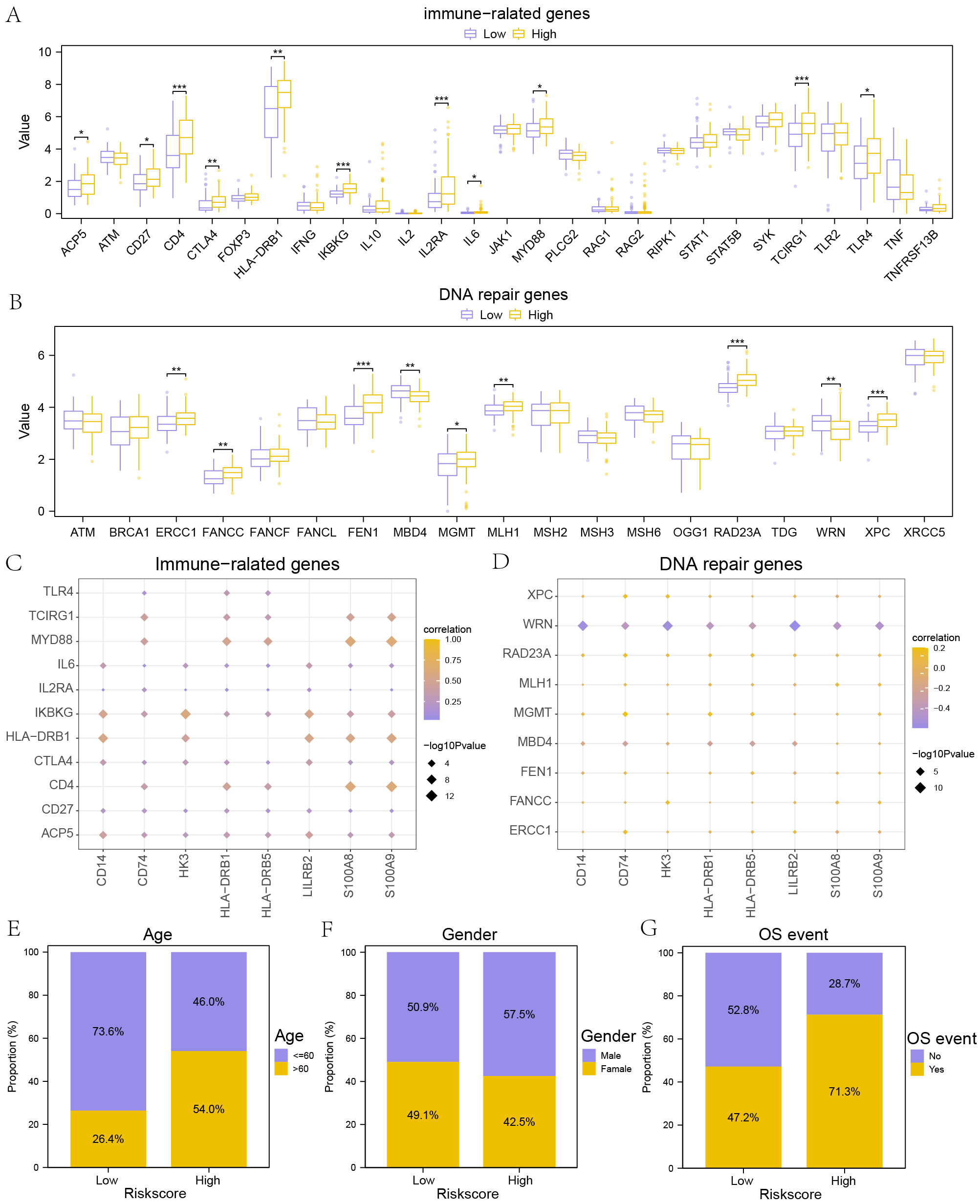

Supplement: Supplementary Figure 9 — Difference analysis between high- and low-risk groups. A, B. Expression of immune-related genes (A) and DNA repair genes (B) in the AML patients. C, D. Relationships between hub genes and immune-related genes (C) and DNA repair genes (D). E-G. Stacked histogram of the proportions of age (E), gender (F), OS event (G). *P-value< 0.05, **P-value< 0.01, ***P-value< 0.001. [file Image_9.tif]
