# Supplementary material for: Integrated bioinformatic analysis of mitochondrial metabolism-related genes in acute myeloid leukemia
Source: Front Immunol. 2023 Apr 17;14:1120670. doi: 10.3389/fimmu.2023.1120670 (PMC10149950; doi:10.3389/fimmu.2023.1120670)
Supplement: Supplementary file 10 [file Table_1.docx]

### Table S1. List of Acute Myeloid Leukemia Information.

|  | **TCGA-LAML** | **GSE12417** | **GSE37642** |
| --- | --- | --- | --- |
| Platform | TCGA | GPL96 | GPL96 |
| Species | Homo sapiens | Homo sapiens | Homo sapiens |
| Tissue | bone marrow or peripheral blood mononuclear cells | bone marrow or peripheral blood mononuclear cells | bone marrow or peripheral blood mononuclear cells |
| Samples in AML group | 151 | 163 | 422 |
| Reference | / | An 86-probe-set gene-expression signature predicts survival in cytogenetically normal acute myeloid leukemia | A 29-gene and cytogenetic score for the prediction of resistance to induction treatment in acute myeloid leukemia |

### Table S2. Mitochondrial metabolism-related genes list.

| Mitochondrial metabolism-related genes | | | | | |
| --- | --- | --- | --- | --- | --- |
| ACAD9 | FOXRED1 | MT-ND2 | NDUFAF6 | POLG | SUCLG1 |
| BCS1L | LIAS | MT-ND3 | NDUFS1 | POLG2 | SURF1 |
| BOLA3 | MPV17 | MT-ND4 | NDUFS2 | SCO1 | TACO1 |
| COX10 | MT-ATP6 | MT-ND5 | NDUFS4 | SCO2 | TIMM8A |
| COX15 | MT-CO2 | MT-ND6 | NDUFS7 | SDHA | TMEM70 |
| DGUOK | MT-CO3 | NDUFA1 | NDUFS8 | SDHD | TRIT1 |
| ECHS1 | MT-CYB | NDUFA13 | NDUFV1 | SLC25A4 | TTC19 |
| ETHE1 | MTFMT | NDUFAF5 | NFU1 | SUCLA2 | TWNK |
| FBXL4 | MT-ND1 |  |  |  |  |

### Table S3. MMRGs Prognosis Model high- and low-risk group phenotype genes list.

| immune-related genes | | DNA repair genes | |
| --- | --- | --- | --- |
| STAT5B | IL2 | ATM | MLH1 |
| CTLA4 | JAK1 | BRCA1 | MSH2 |
| RAG2 | TNFRSF13B | CHK2 | MSH3 |
| RAG1 | TLR2 | ERCC1 | MSH6 |
| FOXP3 | TCIRG1 | FANCC | OGG1 |
| PLCG2 | IKBKG | FANCF | RAD23A |
| IL10 | CD4 | FANCL | TDG |
| ACP5 | HLA-DRB1 | FEN1 | WRN |
| IFNG | STAT1 | MBD4 | XPC |
| TNF | RIPK1 | MGMT | XRCC5 |
| SYK | IL2RA |  |  |
| IL6 | CD27 |  |  |
| ATM | TLR4 |  |  |
| MYD88 |  |  |  |

### Table S4. FAB stages and MM scores of AML patients in TCGA-AML dataset.

| FAB | MM score |
| --- | --- |
| M0 Undifferentiated | 7.441541365 |
| M1 | 7.416364788 |
| M2 | 7.347737208 |
| M2 | 7.471717524 |
| M4 | 7.613878929 |
| M2 | 7.333245517 |
| M4 | 7.359682266 |
| M0 Undifferentiated | 7.492145447 |
| M4 | 7.329780825 |
| M2 | 7.413332057 |
| M2 | 7.288216091 |
| M2 | 7.756806698 |
| M0 Undifferentiated | 7.14538494 |
| M1 | 7.353088337 |
| M0 Undifferentiated | 7.327769067 |
| M3 | 7.521674889 |
| M5 | 7.569859708 |
| M4 | 7.516917713 |
| M4 | 7.378461005 |
| M4 | 7.600225272 |
| M4 | 7.352727663 |
| M5 | 7.642569117 |
| M4 | 7.432046142 |
| M2 | 7.326040283 |
| M3 | 7.35250348 |
| M3 | 7.468170201 |
| M4 | 7.48188125 |
| M6-M7 | 7.613561923 |
| M2 | 7.634526984 |
| M2 | 7.713626201 |
| M4 | 7.484663998 |
| M1 | 7.507133452 |
| M0 Undifferentiated | 6.93753847 |
| M5 | 7.405186618 |
| M1 | 7.533811992 |
| M4 | 7.846361008 |
| M6-M7 | 7.421870823 |
| M2 | 7.589812743 |
| M2 | 7.417025588 |
| M5 | 7.92801992 |
| M3 | 7.542551147 |
| M1 | 7.596403635 |
| M1 | 7.38116804 |
| M1 | 7.616205185 |
| M2 | 7.213760925 |
| M2 | 7.494295109 |
| M1 | 7.402047157 |
| M1 | 7.411734193 |
| M3 | 7.303583491 |
| M5 | 7.330858728 |
| M2 | 7.463270449 |
| M2 | 7.565439518 |
| M2 | 7.46021445 |
| M1 | 7.448111771 |
| M2 | 7.540383763 |
| M1 | 7.558255776 |
| M1 | 7.491583285 |
| M2 | 7.227958324 |
| M5 | 7.596464268 |
| M1 | 7.582421601 |
| M0 Undifferentiated | 7.287917391 |
| M2 | 7.56191974 |
| M4 | 7.477466389 |
| M4 | 7.572090653 |
| M0 Undifferentiated | 7.249068126 |
| M1 | 7.542322557 |
| M4 | 7.417753068 |
| M5 | 7.745708167 |
| M5 | 7.768831816 |
| M1 | 7.395485073 |
| M5 | 7.752809788 |
| M3 | 7.228904335 |
| M1 | 7.228126718 |
| M4 | 7.293549328 |
| M1 | 7.536458677 |
| M2 | 7.478247679 |
| M2 | 7.395037682 |
| M5 | 7.881094729 |
| M4 | 7.279665732 |
| M4 | 7.328433465 |
| M0 Undifferentiated | 7.369153804 |
| M2 | 7.173918035 |
| M2 | 7.460417047 |
| M4 | 6.92801992 |
| M0 Undifferentiated | 7.122000717 |
| M1 | 7.737574309 |
| M1 | 7.501418874 |
| M2 | 7.301603258 |
| M4 | 7.775384107 |
| M4 | 7.514439084 |
| M5 | 7.839277476 |
| M1 | 7.32880109 |
| M1 | 7.418815765 |
| M0 Undifferentiated | 7.421224777 |
| M2 | 7.675739773 |
| M2 | 7.647605937 |
| M5 | 7.557127412 |
| M4 | 7.264900699 |
| M1 | 7.518685994 |
| M4 | 7.47673489 |
| M0 Undifferentiated | 7.300927492 |
| M1 | 7.430205215 |
| M6-M7 | 7.347652783 |
| M1 | 7.432020415 |
| M2 | 7.521877019 |
| M4 | 7.471901142 |
| M1 | 7.389730851 |
| M0 Undifferentiated | 7.423360182 |
| M2 | 7.573692794 |
| M4 | 7.547257369 |
| M0 Undifferentiated | 7.549699613 |
| M2 | 7.569052036 |
| M1 | 7.448504198 |
| M2 | 7.124248401 |
| M5 | 7.790169726 |
| M0 Undifferentiated | 6.992683645 |
| M1 | 7.560043255 |
| M4 | 7.556701254 |
| M2 | 7.331691451 |
| M4 | 7.30966607 |
| M4 | 7.111172713 |
| M4 | 7.207184556 |
| M2 | 7.43823045 |
| M1 | 7.53393064 |
| M2 | 7.663629953 |
| M1 | 7.521885948 |
| M3 | 7.570617789 |
| M5 | 7.5154423 |
| M3 | 7.473929559 |
| M0 Undifferentiated | 7.388204308 |
| M1 | 7.343725149 |
| M2 | 7.744931832 |
| M5 | 7.298823726 |
| M1 | 7.70175332 |
| M1 | 7.740732111 |
| M3 | 7.28771049 |
| M1 | 7.141012237 |
| M3 | 7.181618941 |
| M2 | 7.369717349 |
| M2 | 7.402746677 |
| M3 | 7.441619117 |
| M3 | 7.516687131 |
| M2 | 7.593023305 |
| M3 | 7.310803124 |
| M2 | 7.355040869 |
| M3 | 7.494530258 |
| M1 | 7.509151414 |
| M4 | 7.362445562 |
| M1 | 7.239802193 |
| M3 | 7.295340284 |

### Table S5. Univariate Cox regression analysis to MMRGs associated with OS in TCGA-LAML.

| Characteristics | Total(N) | HR (95% CI) | P value |
| --- | --- | --- | --- |
| BCS1L | 140 | 1.579 (0.957-2.603) | 0.074 |
| COX10 | 140 | 1.223 (0.612-2.444) | 0.569 |
| DGUOK | 140 | 1.847 (0.836-4.078) | 0.129 |
| ECHS1 | 140 | 2.424 (1.516-3.877) | <0.001 |
| ETHE1 | 140 | 0.946 (0.629-1.422) | 0.788 |
| MPV17 | 140 | 1.128 (0.500-2.541) | 0.772 |
| NDUFA1 | 140 | 1.432 (0.990-2.070) | 0.056 |
| NDUFA13 | 140 | 1.160 (0.735-1.832) | 0.524 |
| NDUFS2 | 140 | 6.848 (3.094-15.157) | <0.001 |
| NDUFS7 | 140 | 1.545 (0.918-2.602) | 0.102 |
| NDUFV1 | 140 | 2.194 (1.356-3.552) | 0.001 |
| POLG | 140 | 2.396 (1.177-4.877) | 0.016 |
| SDHA | 140 | 4.568 (2.560-8.152) | <0.001 |
| SUCLG1 | 140 | 5.369 (2.243-12.852) | <0.001 |
| SURF1 | 140 | 1.657 (0.868-3.163) | 0.126 |
| TACO1 | 140 | 2.786 (1.053-7.376) | 0.039 |
| TIMM8A | 140 | 0.973 (0.502-1.885) | 0.934 |

### Table S6. GO enrichment analysis results of MMRGs Prognosis Model high- and low-risk group DEGs.

| Ontology | ID | Description | GeneRatio | BgRatio | pvalue | p.adjust | qvalue |
| --- | --- | --- | --- | --- | --- | --- | --- |
| BP | GO:0019882 | antigen processing and presentation | 10/36 | 226/18670 | 1.07e-11 | 1.57e-08 | 1.01e-08 |
| BP | GO:0001819 | positive regulation of cytokine production | 12/36 | 464/18670 | 3.51e-11 | 2.59e-08 | 1.65e-08 |
| BP | GO:0002283 | neutrophil activation involved in immune response | 11/36 | 488/18670 | 1.16e-09 | 3.52e-07 | 2.25e-07 |
| BP | GO:0002446 | neutrophil mediated immunity | 11/36 | 499/18670 | 1.46e-09 | 3.52e-07 | 2.25e-07 |
| BP | GO:0042110 | T cell activation | 10/36 | 464/18670 | 1.16e-08 | 1.71e-06 | 1.09e-06 |
| CC | GO:0030666 | endocytic vesicle membrane | 10/38 | 167/19717 | 5.58e-13 | 3.63e-11 | 1.91e-11 |
| CC | GO:0030139 | endocytic vesicle | 11/38 | 303/19717 | 7.84e-12 | 2.55e-10 | 1.34e-10 |
| CC | GO:0005765 | lysosomal membrane | 11/38 | 354/19717 | 4.18e-11 | 6.22e-10 | 3.27e-10 |
| CC | GO:0098852 | lytic vacuole membrane | 11/38 | 355/19717 | 4.30e-11 | 6.22e-10 | 3.27e-10 |
| CC | GO:0005774 | vacuolar membrane | 11/38 | 412/19717 | 2.10e-10 | 2.46e-09 | 1.29e-09 |
| MF | GO:0042277 | peptide binding | 11/34 | 295/17697 | 4.67e-12 | 5.79e-10 | 3.54e-10 |
| MF | GO:0033218 | amide binding | 11/34 | 356/17697 | 3.54e-11 | 2.20e-09 | 1.34e-09 |
| MF | GO:0042605 | peptide antigen binding | 5/34 | 31/17697 | 3.16e-09 | 1.30e-07 | 7.97e-08 |
| MF | GO:0003823 | antigen binding | 7/34 | 160/17697 | 1.90e-08 | 5.88e-07 | 3.59e-07 |
| MF | GO:0023023 | MHC protein complex binding | 4/34 | 25/17697 | 1.40e-07 | 3.46e-06 | 2.12e-06 |

### Table S7. KEGG enrichment analysis results of MMRGs Prognosis Model high- and low-risk group DEGs.

| Ontology | ID | Description | GeneRatio | BgRatio | pvalue | p.adjust | qvalue |
| --- | --- | --- | --- | --- | --- | --- | --- |
| KEGG | hsa04640 | Hematopoietic cell lineage | 8/29 | 99/8076 | 1.33e-09 | 6.18e-08 | 4.41e-08 |
| KEGG | hsa04145 | Phagosome | 7/29 | 152/8076 | 8.03e-07 | 9.34e-06 | 6.66e-06 |
| KEGG | hsa04612 | Antigen processing and presentation | 6/29 | 78/8076 | 2.66e-07 | 5.64e-06 | 4.02e-06 |
| KEGG | hsa04658 | Th1 and Th2 cell differentiation | 5/29 | 92/8076 | 1.64E-05 | 9.48E-05 | 6.76E-05 |
| KEGG | hsa05310 | Asthma | 6/29 | 31/8076 | 8.55e-10 | 6.18e-08 | 4.41e-08 |
| KEGG | hsa05330 | Allograft rejection | 5/29 | 38/8076 | 1.92e-07 | 5.64e-06 | 4.02e-06 |
| KEGG | hsa05332 | Graft-versus-host disease | 5/29 | 42/8076 | 3.22e-07 | 5.64e-06 | 4.02e-06 |
| KEGG | hsa04940 | Type I diabetes mellitus | 5/29 | 43/8076 | 3.64e-07 | 5.64e-06 | 4.02e-06 |
| KEGG | hsa04672 | Intestinal immune network for IgA production | 5/29 | 49/8076 | 7.10e-07 | 9.34e-06 | 6.66e-06 |
| KEGG | hsa05150 | Staphylococcus aureus infection | 6/29 | 96/8076 | 9.18e-07 | 9.49e-06 | 6.77e-06 |
| KEGG | hsa05320 | Autoimmune thyroid disease | 5/29 | 53/8076 | 1.06e-06 | 9.84e-06 | 7.02e-06 |
| KEGG | hsa05416 | Viral myocarditis | 5/29 | 60/8076 | 1.98e-06 | 1.67e-05 | 1.19e-05 |
| KEGG | hsa05152 | Tuberculosis | 7/29 | 180/8076 | 2.51e-06 | 1.94e-05 | 1.39e-05 |
| KEGG | hsa05321 | Inflammatory bowel disease | 5/29 | 65/8076 | 2.96e-06 | 2.11e-05 | 1.51e-05 |
| KEGG | hsa04514 | Cell adhesion molecules | 6/29 | 149/8076 | 1.19e-05 | 7.40e-05 | 5.27e-05 |
| KEGG | hsa05168 | Herpes simplex virus 1 infection | 6/29 | 498/8076 | 0.008 | 0.027 | 0.019 |

### Table S8. DO enrichment analysis results of MMRGs Prognosis Model high- and low-risk group DEGs.

| ID | Description | GeneRatio | BgRatio | p.adjust | qvalue |
| --- | --- | --- | --- | --- | --- |
| DOID:2789 | parasitic protozoa infectious disease | 7/30 | 127/8007 | 0.00013 | 7.52E-05 |
| DOID:1398 | parasitic infectious disease | 7/30 | 150/8007 | 0.00020 | 0.00012 |
| DOID:12365 | malaria | 6/30 | 103/8007 | 0.00022 | 0.00013 |
| DOID:2377 | multiple sclerosis | 7/30 | 169/8007 | 0.00022 | 0.00013 |
| DOID:3213 | demyelinating disease | 7/30 | 175/8007 | 0.00022 | 0.00013 |
| DOID:865 | vasculitis | 6/30 | 122/8007 | 0.00032 | 0.00019 |
| DOID:9008 | psoriatic arthritis | 3/30 | 10/8007 | 0.00032 | 0.00019 |
| DOID:850 | lung disease | 10/30 | 499/8007 | 0.00039 | 0.00023 |
| DOID:11335 | sarcoidosis | 5/30 | 84/8007 | 0.00058 | 0.00034 |
| DOID:2916 | hypersensitivity reaction type IV disease | 5/30 | 88/8007 | 1.64E-05 | 0.00065 |

### Table S9. GSEA enrichment analysis results of MMRGs prognosis model high- and low-risk group genes.

| Description | setSize | enrichmentScore | NES | p.adjust | qvalues |
| --- | --- | --- | --- | --- | --- |
| GAL_LEUKEMIC_STEM_CELL_DN | 230 | 0.649679 | 1.903991 | 1.31E-08 | 9.97E-09 |
| BIOCARTA_INFLAM_PATHWAY | 27 | 0.759194 | 1.814192 | 0.00079 | 0.00060 |
| WP_EBOLA_VIRUS_INFECTION_IN_HOST | 129 | 0.619987 | 1.766559 | 1.33E-06 | 1.01E-06 |
| ROSS_LEUKEMIA_WITH_MLL_FUSIONS | 79 | 0.636965 | 1.748821 | 6.74E-05 | 5.11E-05 |
| REACTOME_MITOCHONDRIAL_TRANSLATION | 94 | 0.614458 | 1.713407 | 0.00017 | 0.00013 |
| MOOTHA_GLYCOLYSIS | 21 | 0.757021 | 1.707640 | 0.00516 | 0.00392 |
| KESHELAVA_MULTIPLE_DRUG_RESISTANCE | 76 | 0.617568 | 1.687500 | 0.00073 | 0.00055 |
| VERHAAK_AML_WITH_NPM1_MUTATED_UP | 183 | 0.792164 | 2.300600 | 1.31E-08 | 9.97E-09 |
| VALK_AML_CLUSTER_5 | 31 | 0.869124 | 2.116963 | 4.97E-08 | 3.77E-08 |
| ICHIBA_GRAFT_VERSUS_HOST_DISEASE_35D_UP | 143 | 0.731134 | 2.094350 | 1.31E-08 | 9.97E-09 |
| ICHIBA_GRAFT_VERSUS_HOST_DISEASE_D7_UP | 117 | 0.740114 | 2.089031 | 1.31E-08 | 9.97E-09 |
| VALK_AML_CLUSTER_15 | 30 | 0.844472 | 2.055237 | 5.48E-07 | 4.16E-07 |
| MCLACHLAN_DENTAL_CARIES_UP | 238 | 0.697618 | 2.044344 | 1.31E-08 | 9.97E-09 |
| NAKAYAMA_SOFT_TISSUE_TUMORS_PCA1_UP | 75 | 0.747828 | 2.040606 | 1.31E-08 | 9.97E-09 |
| KEGG_AUTOIMMUNE_THYROID_DISEASE | 50 | 0.779006 | 2.035689 | 1.00E-07 | 7.62E-08 |

### Table S10. mRNA-RBP interaction network nodes.

| mRNA |  | RBP |  | mRNA |  | RBP |
| --- | --- | --- | --- | --- | --- | --- |
| CD14 | - | SND1 |  | CD74 | - | TAF15 |
| CD74 | - | ADAR |  | CD74 | - | TIA1 |
| CD74 | - | AUH |  | CD74 | - | TRA2A |
| CD74 | - | BCCIP |  | CD74 | - | U2AF1 |
| CD74 | - | BUD13 |  | CD74 | - | U2AF2 |
| CD74 | - | CSTF2T |  | CD74 | - | YTHDC1 |
| CD74 | - | DGCR8 |  | HK3 | - | DGCR8 |
| CD74 | - | EWSR1 |  | HK3 | - | EIF4A3 |
| CD74 | - | FAM120A |  | HK3 | - | FUS |
| CD74 | - | FBL |  | HK3 | - | HNRNPL |
| CD74 | - | FUS |  | HK3 | - | NOP58 |
| CD74 | - | GTF2F1 |  | HK3 | - | RBM10 |
| CD74 | - | HNRNPA1 |  | HK3 | - | TAF15 |
| CD74 | - | HNRNPK |  | HK3 | - | TARDBP |
| CD74 | - | HNRNPL |  | HLA-DRB1 | - | FUS |
| CD74 | - | HNRNPUL1 |  | HLA-DRB1 | - | IGF2BP2 |
| CD74 | - | IGF2BP1 |  | HLA-DRB1 | - | IGF2BP3 |
| CD74 | - | IGF2BP2 |  | HLA-DRB1 | - | MOV10 |
| CD74 | - | IGF2BP3 |  | HLA-DRB1 | - | NOP58 |
| CD74 | - | LARP7 |  | HLA-DRB1 | - | TAF15 |
| CD74 | - | LIN28A |  | HLA-DRB1 | - | TARDBP |
| CD74 | - | LIN28B |  | HLA-DRB5 | - | FUS |
| CD74 | - | MBNL2 |  | HLA-DRB5 | - | NOP58 |
| CD74 | - | NOP58 |  | HLA-DRB5 | - | TAF15 |
| CD74 | - | PCBP2 |  | LILRB2 | - | ADAR |
| CD74 | - | PRPF8 |  | LILRB2 | - | HNRNPA1 |
| CD74 | - | RBFOX2 |  | LILRB2 | - | HNRNPL |
| CD74 | - | SF3A3 |  | LILRB2 | - | IGF2BP3 |
| CD74 | - | SF3B4 |  | LILRB2 | - | SRSF1 |
| CD74 | - | SMNDC1 |  | LILRB2 | - | TAF15 |
| CD74 | - | SND1 |  | LILRB2 | - | U2AF2 |
| CD74 | - | SRSF1 |  | S100A8 | - | ADAR |
| CD74 | - | SRSF10 |  | S100A9 | - | HNRNPL |
| CD74 | - | SRSF7 |  | S100A9 | - | SRSF3 |
| CD74 | - | SRSF9 |  |  |  |  |

“mRNA” and “RBP” represent node; “-” represent edge; RBP, RNA binding protein.

### Table S11. mRNA-drugs interaction network nodes.

| mRNA |  | drug |  | mRNA |  | drug |
| --- | --- | --- | --- | --- | --- | --- |
| CD14 | - | IC14 |  | HLA-DRB1 | - | PRAVASTATIN |
| CD14 | - | LOVASTATIN |  | HLA-DRB1 | - | FLUPIRTINE |
| CD74 | - | MILATUZUMAB |  | HLA-DRB1 | - | DAPSONE |
| HLA-DRB1 | - | FLUVASTATIN |  | HLA-DRB1 | - | DABRAFENIB |
| HLA-DRB1 | - | AZATHIOPRINE |  | HLA-DRB1 | - | PROPYLTHIOURACIL |
| HLA-DRB1 | - | EFAVIRENZ |  | HLA-DRB1 | - | SIMVASTATIN |
| HLA-DRB1 | - | ASPARAGINASE |  | HLA-DRB1 | - | PITAVASTATIN |
| HLA-DRB1 | - | TICLOPIDINE |  | HLA-DRB1 | - | AMOXICILLIN |
| HLA-DRB1 | - | ETANERCEPT |  | HLA-DRB1 | - | LUMIRACOXIB |
| HLA-DRB1 | - | OXCARBAZEPINE |  | HLA-DRB1 | - | LAPATINIB |
| HLA-DRB1 | - | ADALIMUMAB |  | HLA-DRB1 | - | CLAVULANIC ACID |
| HLA-DRB1 | - | INFLIXIMAB |  | HLA-DRB1 | - | LAMOTRIGINE |
| HLA-DRB1 | - | LYM-1 |  | HLA-DRB1 | - | FLOXACILLIN |
| HLA-DRB1 | - | BUCILLAMINE |  | HLA-DRB5 | - | CLAVULANIC ACID |
| HLA-DRB1 | - | CARBIMAZOLE |  | HLA-DRB5 | - | AMOXICILLIN |
| HLA-DRB1 | - | ROSUVASTATIN |  | HLA-DRB5 | - | 1D09C3 |
| HLA-DRB1 | - | CARBAMAZEPINE |  | HLA-DRB5 | - | CLOZAPINE |
| HLA-DRB1 | - | MERCAPTOPURINE |  | S100A8 | - | METHOTREXATE |
| HLA-DRB1 | - | ASPIRIN |  | S100A9 | - | TASQUINIMOD |
| HLA-DRB1 | - | METHIMAZOLE |  | S100A9 | - | PAQUINIMOD |
| HLA-DRB1 | - | ATORVASTATIN |  |  |  |  |

“mRNA” and “drug” represent node; “-” represent edge.
